# Resiliency of Environmental and Social Stocks: An Analysis of the Exogenous COVID-19 Market Crash

**DOI:** 10.1093/rcfs/cfaa011

**Published:** 2020-07-07

**Authors:** Rui Albuquerque, Yrjo Koskinen, Shuai Yang, Chendi Zhang

**Affiliations:** 1 Carroll School of Management, Boston College, ECGI, and CEPR; 2 Haskayne School of Business, University of Calgary; 3 University of Exeter Business School

**Keywords:** G12, G32, M14

## Abstract

The COVID-19 pandemic and the subsequent lockdown brought about an exogenous and unparalleled stock market crash. The crisis thus provides a unique opportunity to test theories of environmental and social (ES) policies. This paper shows that stocks with higher ES ratings have significantly higher returns, lower return volatility, and higher operating profit margins during the first quarter of 2020. ES firms with higher advertising expenditures experience higher stock returns, and stocks held by more ES-oriented investors experience less return volatility during the crash. This paper highlights the importance of customer and investor loyalty to the resiliency of ES stocks.

* We thank the journal's editors Andrew Ellul, Isil Erel, and Uday Rajan; colleagues Alex Edmans, Caroline Flammer, Philipp Krüger, Hao Liang, Pedro Matos, Luc Renneboog, Lukas Roth, Henri Servaes, Philip Strahan, Dragon Tang, Hassan Tehranian, and Alex Wagner; and seminar participants at the University of Exeter and the Canadian Sustainable Finance Network for their helpful comments. An earlier version of this paper circulated under the title "Love in the Time of COVID-19: The Resiliency of Environmental and Social Stocks." Albuquerque benefitted from financial support from the Portuguese Foundation for Science and Technology [PTDC/IIM-FIN/2977/2014]. All remaining errors are our responsibility. Send correspondence to Rui Albuquerque, rui.albuquerque@bc.edu.

The predominant view of socially responsible firms is that they maximize shareholder welfare by engaging in environmental, social, and governance (ESG) activities (e.g., McWilliams and Siegel 2001). This view is often summarized as “doing well by doing good”: ESG activities are good for shareholders, while striving for big social goals. The opposite view on ESG activities is predicated on the notion, usually attributed to Friedman (1970), that those activities are just a manifestation of managerial agency problems between shareholders and managers. In this view, managers engage in ESG activities that will generate benefits to them at the expense of shareholders.[1]

For quite some time now, practitioners have taken the view that ESG activities create value for firms and their shareholders. For example, McKinsey’s 2019 Global Survey on ESG programs reports that a large majority of executives and investment professionals agree that ESG policies increase shareholder value (McKinsey & Company 2020). The same was true already in their 2009 survey. The academic literature has shown a positive association between ESG and financial performance.[2] The difficulty, though, lies in identifying the direction of causality and the underlying mechanisms: is it the case that firms with strong financial performance can afford to engage in ESG activities, or is it that ESG activities add value to shareholders? This paper addresses the empirical challenge by positing that the COVID-19 pandemic is an exogenous shock that allows us to study the causal link from ESG to financial performance.[3]

We argue that the COVID-19 pandemic presents an unparalleled shock. First, the COVID-19 crisis and the subsequent economic lockdown is an unexpected shock to global stock markets. Second, it is an exogenous shock that originated out of public health concerns, not because of economic conditions. Third, the pandemic resulted in a stock market crash. The stock market in the United States peaked on February 19, and a mere month later prices had declined by almost 30%. The unexpected and exogenous nature of the shock and its speed suggest that firms had very limited ability to respond in a timely fashion to the unfolding crisis. Thus, the stock market reacted mostly to firms’ preexisting conditions that affected their ability to endure the crisis. Overall, these aspects of the crisis create the opportunity for an event study that uses a very narrow window of time to test the causal link between ESG and firm value.

1 Benabou and Tirole (2010) discuss three possible views of corporate ESG activities: ESG activities motivate firms to adopt a longer-term perspective; ESG activities are delegated prosocial behaviors; and ESG activities are insider-initiated corporate philanthropy.

2 See, for example, the meta-analyses of ESG activities and financial performance by Orlitzky, Schmidt, and Rynes (2003), Margolis, Elfenbein, and Walsh (2010), and Busch and Friede (2018).

3 The *Financial Times* Alphaville column (April 2, 2020) labels the COVID-19 pandemic as the “ESG acid test” (Powell 2020).

To understand why the COVID-19 shock is useful to study the ESG-financial performance link, consider the following two theories of ESG activities based on customer and investor preferences. Albuquerque, Koskinen, and Zhang (2019) present a model where firms invest in ESG policies as a product differentiation strategy (e.g., Patagonia uses only organic cotton in its outdoor clothing and supports conservation efforts; Apple is switching to 100% renewable energy; and TOMS donates a pair of shoes for every pair bought). The benefit of this strategy is a more loyal customer base and a lower price-elasticity of demand for their products. A less price-elastic demand gives the firm the ability to charge higher prices and have higher profit margins. In their model, the higher profit margin lowers operating leverage and thus systematic risk, and increases firm value. If the COVID-19 shock affects consumer demand, customer loyalty for ESG firms is hypothesized to benefit ESG firms' stock performance and resiliency.

The literature on sustainable and responsible investments (SRI) provides another hypothesis of how the COVID-19 shock affects the ESG-financial performance link. This literature has shown that ESG investors—investors with a preference for ESG stocks—are less sensitive to SRI funds' performance relative to conventional mutual funds' performance (Bollen 2007; Renneboog, Ter Horst, and Zhang 2011).[4] If the COVID-19 shock affects an investor's attitude toward risk, with many investors selling their holdings, the SRI literature suggests that ESG investors are more resilient compared to investors in other stocks.[5] Heinkel, Kraus, and Zechner (2001) develop a model of segmented capital markets based on investor preferences where a polluting firm, held by only a subset of investors, is less diversified and therefore carries greater systematic risk relative to other firms. Consequently, green firms, arguably firms with high ESG ratings, have higher valuations. If the COVID-19 shock led investors to flee the market, but less so for those ESG investors, then the price of ESG stocks should not decline as much, relative to the price of other stocks.

These two theories predict that stocks with high ESG ratings are more resilient relative to other stocks in the rampant stock market sell-off during the first quarter of 2020. Each theory offers a specific, though not necessarily mutually exclusive, mechanism that we also test in this paper.

We focus on the environmental and social (ES) aspects of ESG to avoid capturing a governance effect. Consistent with the paper's main prediction, our first result is that first quarter abnormal returns are significantly correlated with ES ratings in the cross-section, even after controlling for the usual firm

4 Using data from Morningstar on the sustainability of mutual funds to explore how fund investments are allocated, Hartzmark and Sussman (2019) show evidence that investors value sustainability due to nonfinancial motives and biases in performance expectations.

5 In fact, the *Financial Times* reports increasing fund flows into ESG ETFs at the same time that conventional equity ETFs experience declining inflows and even outflows in the United States (Tett et al. 2020).

characteristics, including size, cash to assets, Tobin's q, dividend yield, volatility, leverage, and industry. Next, we examine more closely the relation between the returns for firms with high ES ratings and the COVID-19 pandemic by using daily data and conducting a difference-in-differences analysis inside the first quarter of 2020. We estimate a difference-in-differences regression of firm-level daily abnormal returns with a COVID-19 event date of February 24,[6] when the stock market decline accelerated. We include a second event date of March 18, when President Trump signed the second Coronavirus Emergency Aid Package, which is the start of an aggressive fiscal and monetary policy response to the pandemic. We control for the second event because we wish to have a cleaner identification of the effect of the COVID-19 pandemic. We add firm and day fixed effects to control for any other unobservable effects, and cluster the standard errors by firm and day. We find that firms with high ES ratings earn an extra daily return of 0.45% from February 24 until March 17 relative to firms with low ES ratings, for a cumulative difference of 7.2%. We conduct a formal test of parallel trends, and do not reject the parallel trends assumption.

We complement the difference-in-differences regressions with a less parametric study of the relation between the returns to ES ratings and the COVID-19 pandemic. Following Ramelli and Wagner (forthcoming), we estimate daily cross-sectional regressions of cumulative abnormal returns of U.S.-listed firms and inspect the evolution of the loading on ES ratings over time. We find that the loading on ES ratings is flat from January 1, 2020, until the end of February, which suggests no significant return difference between high- and low-ES firms prior to the COVID-19 shock and, as a by-product, supports the parallel trends assumption. The loading on ES ratings then steadily increases until it plateaus around mid-March, consistent with ES stocks being more resilient during the COVID-19 market crash.

Consistent with the resiliency hypothesis, we also document that high ES-rated firms display lower volatility of stock returns during the first quarter of 2020. We do this two ways. First, we compute the standard deviation of daily log returns, raw and capital asset pricing model (CAPM) adjusted, for the first quarter of 2020 and use cross-sectional regression models to study the effect of ES policies on volatility. Second, we use a range-based volatility measure (the daily high price minus the daily low price divided by the average price) and estimate difference-in-differences regressions using daily data. We find that volatility is lower for highly rated ES firms under both approaches and for the various measures of volatility.

6 The S&P 500 peaked on February 19, 2020. On Friday, February 21, several municipalities in Northern Italy entered lockdown, and the subsequent decline in the S&P 500 accelerated.

Next, we study the operating performance of firms with high ES ratings relative to other firms during the first quarter of 2020. In contrast to stock returns, accounting numbers will take some time to fully reflect the worsening economic situation and firms' response to it. This analysis is thus just a first step to a more in depth study as additional data become available. We find that firms with high ES ratings realize higher operating profit margins in the first quarter of 2020 relative to the last quarter of 2019 viz-à-viz other firms, consistent with predictions from Albuquerque, Koskinen, and Zhang (2019). We also find that asset turnover (i.e., ratio of sales to assets) is lower for firms with high ES ratings relative to other firms during the period. High ES firms appear to have been able to increase their margins even as sale proceeds declined. Finally, we find no difference in return on assets for firms with high ES ratings relative to other firms during the first quarter of 2020.

To answer the question of how ES policies help build resiliency, we further investigate the two theories of customer and investor loyalty presented above. In Albuquerque, Koskinen, and Zhang (2019), ES is a product differentiation strategy. Since some markets are more competitive than others, we use advertising expenditures as a way to capture firms' ability to acquire customer loyalty. We therefore expect stronger results for firms with both high ES and advertising expenditures. We show that the effect on stock returns is twice as large for firms with high ES ratings coupled with high advertising expenditures compared to firms with high ES ratings but low advertising. This evidence is consistent with prior research (Servaes and Tamayo 2013; Albuquerque, Koskinen, and Zhang 2019). To test the investor loyalty mechanism, we construct a variable that measures the ES preferences of institutional investors. If firms with high ES ratings have owners with a preference for those stocks, then these firms should perform relatively better during a market sell-off. We find a positive, but insignificant effect of investor preferences on stock returns. Economically, the effect from investor preferences is about half the size of the effect from advertising expenditures. We note that these results are obtained in difference-in-differences regressions where we include firm and day fixed effects to control for unobserved constant effects, and also cluster standard errors by firm and day.

We also test the ability of these variables to explain the changes in the volatility of stock returns. We find a strong negative effect of investor preferences on range-based volatility in firms with high ES ratings. In contrast, our results show a negative, but insignificant effect on volatility for firms with high ES ratings coupled with high advertising expenditures. Overall, our evidence suggests that both mechanisms affect the return performance of high ES firms, relative to other firms (consistent with the findings in Gantchev, Giannetti, and Li 2019). Customer loyalty, however, is a more important factor in explaining the level of stock returns, besides being consistent with the operating profit margin results, whereas investor ES preferences is a more important factor for the volatility of stock returns.

Because of ESG ratings disagreements between different rating agencies (e.g. Berg, Koelbel, and Rigobon 2020), we use ES ratings from Thomson Reuters Refinitiv for our main results, but we find similar results using MSCI ES scores. One alternative explanation for our main finding is that the oil price decline in the first quarter of 2020 affected particularly firms in the energy sector, which are known to score low in some dimensions of ES. We repeat the analysis excluding firms in the energy sector from our sample. We find even stronger results. Another alternative explanation is that some businesses were considered "essential" and kept on operating in a normal fashion. We show that the documented resiliency of high ES-rated firms applies also within each industry, ruling out the essential-firms argument. It is also plausible that our results are driven by corporate governance, since Ferrell, Liang, and Renneboog (2016) show that well-governed firms invest more in ES policies. However, we show that our results for ES stocks cannot be explained by a good corporate governance effect.

Stocks with high ES ratings were not the only stocks to perform better during the first quarter of 2020. Acharya and Steffen (forthcoming) provide evidence that firms with access to liquidity perform better during the first quarter. Ramelli and Wagner (forthcoming) show that nonfinancial firms with higher cash holdings and lower financial leverage are less affected than other firms. Similar evidence is also provided by Fahlenbrach, Rageth, and Stulz (2020). Alfaro et al. (2020) and Hassan et al. (2020) show that stocks that are less exposed to the COVID-19 pandemic perform better. Pagano, Wagner, and Zechner (2020) demonstrate that firms that are less affected by social distancing have higher returns during the crisis. Landier and Thesmar (2020) demonstrate that changes in analysts' forecasts about future corporate earnings explain the overall decline, but not the short-term price movements, in stock prices during COVID-19. Shan and Tang (2020) document that Chinese firms with greater employee satisfaction appear to endure the COVID-19 stock market downturn better than other firms, supporting employee satisfaction as one dimension of ES policies creating shareholder value (Edmans 2011). In a cross-country analysis, Ding et al. (2020) provide evidence that firms with stronger balance sheets, less exposure to COVID-19, and more sustainable operations perform better during the first quarter. Cheema-Fox et al. (2020) show that firms that protect their workforce and supply chains during the stock market collapse have higher returns than other firms.

In addition to affecting stock prices, COVID-19 dramatically affected corporate financing. Li, Strahan, and Zhang (forthcoming) document an unprecedented increase in commercial and industrial loans in banks' balance sheets, as nonfinancial corporations draw funds from credit lines during the three last weeks in March. Halling, Yu, and Zechner (forthcoming) present evidence that bond issuance increases significantly after the middle of March, especially for highly rated bonds. Firms choose to issue bonds with longer maturities, perhaps anticipating that cash flows will be low for a long time.

Several recent papers have asserted a positive causal link from ESG activities to firms' financial performance. El Ghoul et al. (2011) employ instrumental variables estimation and dynamic panel data methods to show causality from ESG activities to lower cost of capital. Albuquerque, Koskinen, and Zhang (2019) similarly use instrumental variables estimation to demonstrate a causal link from ESG to reduced systematic risk and increased valuations. Dimson, Karakas, and Li (2015) and Krüger (2015) use event-study analyses to link ESG events to subsequent firm financial performance; their method alleviates concerns about reverse causality and omitted variables. Flammer (2015) employs the regression discontinuity design to show that successful shareholder ESG proposals result in positive abnormal returns. Masulis and Reza (2015), however, use the 2003 Tax Reform Act, which reduced personal tax rates on dividends, as an exogenous event to show that corporate giving—a component of ESG policies—reduces shareholder wealth. Their findings support the agency costs viewpoint.

In their paper studying the Great Recession of 2008–2009, a major economic shock, Lins, Servaes, and Tamayo (2017) show that U.S. nonfinancial firms with high ES ratings had better financial performance than other firms.[7] The current crisis is very different from the Great Recession for the speed and nature of the shock. In the 2-year duration of the Great Recession, firms had plenty of opportunities to adjust to the crisis and new government policies.[8] Thus, the Great Recession is a noisier setting in which to identify the effect of ESG on stock market performance because of the length of the economic shock. Second, the current shock is an unpredictable public health shock that is exogenous to the U.S. economy. In contrast, the Great Recession was economically driven and its origins in the financial sector led to widely held mistrust for financial firms. A confounding effect between ES policies and trust potentially limits our ability to discern whether the good performance of firms with high ES ratings in 2007–2008 is attributable to ES policies or to trust in general.

## 1. Data and Methodology

### 1.1 Sample and summary statistics

Our main data source on firms' ES performance is Thomson Reuters' Refinitiv ESG database. We include all U.S. stocks in the Refinitiv database. Refinitiv collects information from corporate annual reports, sustainability reports, nongovernmental organizations, and news sources for publicly traded companies at an annual frequency. Refinitiv ESG evaluates firms' environmental (E) performance in three categories: resource use, emissions, and innovation. Social (S) commitments are measured in four

7 Cornett, Erhemjamts, and Tehranian (2016) show that U.S. banks' financial performance during the Great Recession is positively related to their ESG score.

8 Dai, Rau, and Tan (2020) demonstrate that firms increase their ESG scores during times of heightened uncertainty about economic policy conditions.

areas: workplace, human rights, community, and product responsibility. Governance (G) is evaluated in three dimensions: management, shareholders, and corporate social responsibility strategy. Each subcategory contains several ESG themes. For example, the resource use category contains four themes: water, energy, sustainable packaging, and environmental supply chain. The emission category covers themes of $CO_2$ emissions, waste, biodiversity, and environmental management systems. The ESG subcategory on workforce includes four themes: diversity and inclusion; career development and training; working conditions; and health and safety. The scores are based on the relative performance and materiality of ESG factors within the firm's sector (for E and S) and country (for G) and range from 0 to 100. Thomson Reuters' Refinitiv ESG scores have been used in the prior literature (e.g., Ferrell, Liang, and Renneboog 2016; Dyck et al. 2019). Our main measure, ES, is the average of the environment and social scores in 2018, expressed as a percentage. We thus omit the governance score.

We obtain daily stock returns from Capital IQ North America Daily for the first quarter of 2020 and CRSP from 2017 to 2019. The daily abnormal return is estimated as the difference between the daily logarithm return (i.e., the logarithm of gross return) of a stock and the CAPM beta times the daily logarithm return of the market.[9] The CAPM beta is estimated using daily returns from 2017 and 2019, and the S&P 500 as the market index. Similarly, the quarterly abnormal return is the difference between the logarithm of the stock's gross quarterly return and the CAPM beta times the logarithm of the market's gross quarterly return. We then calculate the volatility of stock returns, both raw and CAPM adjusted.

Accounting data for 2019 are obtained from Compustat and are used to construct control variables, namely, *Tobin's q*, *Size*, *Cash*, *Leverage*, *Return on equity*, *Advertising*, and *Dividend yield*. We winsorize all accounting variables at the 1% level in each tail. Table A1 in the appendix defines all variables used in the paper. After matching all data sets, our sample consists of 134,689 firm-day return observations for 2,171 distinct firms. Table 1 presents summary statistics.

Insert Table 1 here.

We construct a firm-level investor ES measure based on institutional investors revealed preferences. Investors' ES preference is estimated using institutional investors' equity holdings, following recent studies (Starks, Venkat, and Zhu 2018; Gibson et al. 2019). We measure institutional ownership using Thomson Reuters' 13F database, which reports institutional investors' equity holdings. We merge the 13F investor holding data with Refinitiv ESG data for U.S. stocks. To construct the measure, we first measure an investor's ES preference as the value-weighted average Refinitiv ES score of its portfolio

9 Our results are similar if we use arithmetic returns instead.

holdings for each quarter in 2018 and then average across the four quarters.[10] Investor-based ES score of a firm is measured as the weighted average of its investors' ES preference based on first quarter of 2019 holdings. We construct the measure for 2,123 stocks in the Refinitiv ESG database where the 13F investor holding data is available.

#### 1.2 Empirical design

To study the effect of ES on corporate financial performance, we run two sets of regressions. Our main set of results uses difference-in-differences regression specifications to better identify the effect of the COVID-19 pandemic. We also use cross-sectional regressions of firms' quarterly stock market performance. The cross-sectional regressions provide less clean estimates of the effect of the crisis, because of the fiscal response that ensued, but provides some external validity by not being tied to a specific shock date. Also, the cross-sectional regressions are comparable with the operating performance regressions for which we only have quarterly data.

Consider first the cross-sectional regression specification:

$$Performance_i = ß_0 + ß_1 ES_i + ß_2 Firm\ controls_i + ß_3 Industry\ FE_i + \varepsilon_i. \quad (1)$$

We use this specification to study the behaviour of three different dependent variables: quarterly abnormal returns, return volatility (total and idiosyncratic volatility), and operating performance (measured by return on assets, operating profit margin, and asset turnover). The unit of observation is firm *i* during the first quarter of 2020. The independent variable of interest, *ES*, is the environmental and social rating of firm *i* in 2018. We control for several firm characteristics. For stock return and volatility regressions, we control for *Tobin's q*, *Size*, *Cash*, *Leverage*, *Return on equity*, *Advertising*, *Historical volatility*, and *Dividend yield* of firm *i* in 2019, and use ordinary least squares. For operating performance regressions, we control for *Tobin's q*, *Cash*, and *Leverage* of firm *i* in 2019, and use median regressions, following Gompers, Ishii, and Metrick (2003). We run regression specifications with and without industry fixed effects based on the Fama and French 12 industry of firm *i*. Standard errors are robust to heteroscedasticity.

[10] An inspection of Table 1 reveals that *Investor-based ES* is much higher than firm *ES*. We have reconstructed an equal-weight investor ES measure. That is, in this new measure, we first equal-weight ES scores of firms held by an investor. This first step is different from our current measure that uses value weights. In a second step, we follow our previous procedure by value-weighting these scores into the firm-level measure. The new measure is much closer to firm-level ES. This is because investor portfolios are tilted to larger firms and larger firms tend to have more ES. The results using this new measure are very similar to our value-weighted measure, suggesting that our results on investors' ES preference are not driven by investor weighting of large cap stocks.

In our main tests of difference-in-differences regressions, we run the following daily regressions:

$$\begin{aligned} \textit{Stock performance}_{it} = \text{ß}_0 + \text{ß}_1 \textit{ES\_treatment}_i \times \textit{Post\_COVID}_t \\ + \text{ß}_2 \textit{ES\_treatment}_i \times \textit{Post\_fiscal}_t + \text{ß}_3 \textit{Firm FE}_i + \text{ß}_4 \textit{Day FE}_t + \varepsilon_{it}. \end{aligned} \quad (2)$$

The two dependent variables we study are daily abnormal returns and daily return volatility (measured by daily price range) of firm *i* on day *t* during the first quarter of 2020. *ES_treatment* is a dummy variable that equals one for firm *i* if its ES rating is ranked in the top quartile in 2018, and zero otherwise. *Post_COVID* equals one from February 24 to March 31, 2020, and zero before this period. *Post_fiscal* equals one from March 18 to March 31, 2020, and zero before this period. We control for the second event to have a cleaner identification of the effect of the COVID-19 pandemic. We include firm and day fixed effects to control for any other unobservable effects, and cluster the standard errors by firm and day.

To understand our choice of event window for *Post_COVID* and *Post_fiscal*, consider Figure 1. Figure 1 depicts S&P 500 performance during the first quarter of 2020, with two dates highlighted: February 24 and March 18, 2020. These dates are used to identify the pandemic shock in our difference-in-differences regressions. February 24 is the start of the “fever” period in Ramelli and Wagner (forthcoming). It is also the first trading day after the first lockdown in Europe, in Northern Italy. We construct a second event dummy to isolate the effect of the U.S. fiscal and monetary policy response to the pandemic on firms’ stock returns. March 18 is the day that President Trump signed the second Coronavirus Emergency Aid Package (CEAP) (the Families First Corona Response Act). March 18 is also the date the Federal Reserve begins making purchases under the Commercial Paper Funding Facility to alleviate the strain in short-term credit markets. The first CEAP signed on March 6 into law is a very small package of $8.3 billion targeted to combat the spread of Coronavirus. The third and largest CEAP (the Coronavirus Aid, Relief, and Economic Security Act) is signed by President Trump on March 27.

Insert Figure 1 here.

In Equation (2), the coefficient on the first interaction term (*ß$_1$)* captures the causal effect of ES policies on stock performance during the crisis, whereas the coefficient on the second interaction term (*ß$_2$)* reflects the additional effect during the second period when we expect the ES effect on stock returns to be weakened by aggressive fiscal and monetary interventions.

To test the specific mechanisms of how ES policies help build resiliency, we add triple interaction terms to the above difference-in-differences regressions. The triple interaction between *ES Treatment*, *Post_COVID*, and a dummy indicating the firms in the top quartile of advertising expenditures (*Investor-based ES*) captures the effect from the customer (investor) loyalty mechanism. We also include a triple interaction with *Post_fiscal* instead of *Post_COVID*. The regressions include all possible double interactions. We continue to include firm and day fixed effects and cluster standard errors by firm and day. We expect that the main effect we capture arises mostly in firms with high customer and investor loyalty.

Our test uses the COVID-19 pandemic shock to detect causality by studying the effect of precrisis ES on financial performance during the crisis, because we measure ES with a lag of more than a year (when the pandemic was unforeseen) and also because in the narrow window during the COVID-19 crisis firms have very little time to respond. Consequently, we attribute the stock market reaction to the predetermined ES policies.

## 2. Results

### 2.1 Level of stock returns

Table 2 presents results of regressing quarterly CAPM-adjusted log returns on firms' ES ratings and other firm characteristics. In column 1, we use ES ratings as the only independent variable. In column 2, we add industry fixed effects, and, in column 3, we add firm controls as independent variables. Standard errors are robust to heteroscedasticity. The effect of ES ratings on stock returns is significant at the 5% level or better, even after controlling for all the variables. The magnitude of the coefficient estimate suggests that one standard deviation increase in ES ratings is associated with a higher stock return in the first quarter of 1.8% on average (8.5 x 0.212). The economic magnitude of this coefficient encompasses the response of ES firms' stock returns both to the pandemic and to the fiscal response at the end of the quarter. Firms with high Tobin's q, larger firms, firms with high cash, firms with lower leverage, firms with lower historical volatility, and firms with lower dividends all perform better (for a discussion of the role of cash and leverage, see Ramelli and Wagner [forthcoming]).

Insert Table 2 here.

To understand the connection between high- and low-rated ES policies and firms' stock returns during the pandemic, consider two pairs of companies: (1) Intel and Broadcom (business equipment industry)

and (2) Verizon and Dish Network (telecommunications industry). Intel and Broadcom ES ratings are 87% and 25%, respectively. Broadcom's ES score is penalized by the risks it faces through its partner companies regarding climate change, hazardous materials, and waste. During the first quarter, Intel's raw return is -9.6% and the CAPM-adjusted return is 17.3%. In contrast, Broadcom's raw return is lower at -25% and its CAPM-adjusted return is 0.4%. A similar picture emerges from Verizon and Dish Network. Verizon's ES rating is 63% with raw returns of 12.5% (-3.6% CAPM-adjusted) during first quarter of 2020, whereas Dish Network's ES rating is lower at 18% with also lower raw returns of -43.6% (-20.8% CAPM-adjusted). Dish Network's ES susceptibility arises from its carbon footprint and pollution management. Of course these firms differ also with respect to size, profitability, and leverage, besides their ES ratings, but for this reason we control for firm characteristics in our cross-sectional regressions.

Next, we conduct a difference-in-differences estimation that captures a tighter link between the performance of firms with high ES ratings and the COVID-19 pandemic by using daily data and two event dummies, *Post_COVID* and *Post_fiscal*. The treatment group of firms is represented by the dummy variable *ES_treatment*. A similar identification strategy is used in Lins, Servaes, and Tamayo (2017), though they do not have the benefit of daily data.

Table 3 contains the results. Column 1 omits fixed effects, and column 2 includes firm and day fixed effects. Standard errors are clustered by firm and day. The results show that the coefficient associated with the interaction between *Post_COVID* and *ES_treatment* is positive and significant at the 1% level. High ES-rated firms earn an average abnormal daily return of 0.45% relative to other firms from February 24 to March 17, for a cumulative effect of 7.2% (0.45% x 16). The economic significance is markedly larger than in the cross-sectional regressions of Table 2, because we are able to identify the response of stock returns to the pandemic with daily data. The results also show that the fiscal response dummy interacted with the high-ES dummy is insignificant. Overall, investors pay more for firms with higher ES ratings as the market collapses in the first quarter of 2020. We perform a test of the parallel trends assumption to establish that our results are not due to diverging behavior of highly rated ES firms relative to other firms even before the COVID-19 pandemic. The Internet Appendix contains a formal test by regressing daily abnormal returns from January 1, 2020, to February 23, 2020, on a dummy for high ES firms and finds an insignificant coefficient. Thus, the difference-in-differences specification satisfies the parallel trends assumption.

To further document the resiliency of stock returns of high ES-rated firms, we conduct daily cross-sectional regressions of cumulative CAPM-adjusted stock returns (from the start of the quarter to each day) on ES ratings, Tobin's q, firm size, cash to assets, financial leverage, return on equity, advertising expenditures, dividend yield, past return volatility, and industry fixed effects (as in Ramelli and Wagner

[forthcoming]). Figure 2 plots the daily loading on ES ratings, cash to assets, and leverage with 90% confidence bands constructed using heteroscedasticity-robust standard errors. The advantage of this analysis relative to the difference-in-differences regressions is that we do not commit to a particular event date to see how the relevancy of ES ratings changes over time. The disadvantage is that it does not give an estimate of the average change in stock returns. The figure shows the loading on ES ratings increasing dramatically at the end of February until it plateaus in mid-March. It describes the building up toward the effect we eventually find in the cross-sectional regressions of quarterly returns (note that the last point estimate in Figure 2 is the same as the point estimate in column 3 of Table 2).[11] Prior to the COVID-19 shock, there is no significant return difference between ES firms and others, consistent with the results from the parallel trends test discussed above. The loading on cash to assets also increases reaching higher levels to that of ES, whereas the loading on leverage is negative and falls precipitously post-February, consistent with Acharya and Steffen (forthcoming) and Ramelli and Wagner (forthcoming). The reasons for the dramatic effect of ES on returns are analyzed in Section 4.

Insert Table 3 and Figure 2 here.

### 2.2 Volatility of stock returns

Toward the resiliency hypothesis of ES firms, we also provide evidence of how the volatility of stock returns varies with ES ratings in the cross-section. In Table 4, we repeat the regressions in Table 2 using the standard deviation of daily raw log returns over the quarter as the dependent variable (columns 1, 2, and 3) and the idiosyncratic volatility calculated as the standard deviation of CAPM-adjusted daily stock returns over the quarter (columns 4, 5, and 6). Standard errors are robust to heteroscedasticity. In all regression specifications, we find that firms with high ES ratings experience a decrease in stock return volatility as compared to other firms (with 1% or better significance level). One standard deviation increase in ES is associated with a decrease in total volatility of 0.29 (-1.374 x 0.212), which represents close to 5% of the mean volatility of stock returns.

Just as with stock returns, we conduct a difference-in-differences analysis to better tie the variation in volatility of stock returns to the COVID-19 pandemic. For this analysis, we use a range-based measure of daily volatility, the daily high price minus the daily low price divided by the average price. We repeat the regressions in Table 3 using price-range volatility as the dependent variable.

[11] During the first quarter of 2020, many high-dividend stocks suspended dividend or share repurchase programs. Based on news headline searches on Factiva, we find that the earliest article with such news is dated March 20 from Dow Jones Newswire. Therefore, this news unlikely affects our results for the first quarter.

Table 5 presents the results. The regressions show that the change in volatility can be traced to the *Post_COVID* treatment variable. Range-based volatility of stock returns for highly rated ES firms decreases relative to other firms. High ES-rated firms experience an average daily decrease in price-range volatility of 0.63% relative to other firms from February 24 to March 17, for a cumulative effect of 10.1% (-0.63% x 16). Similarly to the evidence from stock returns, the economic magnitude of the ES effect increases when the regressions more clearly isolate the effect of the pandemic on the stock market. Table 5 also suggests that the fiscal policy treatment dummy has an added effect contributing to even lower volatility of high ES-rated firm returns relative to other firms.[12]

Overall, the resiliency of high-rated ES stock returns is displayed both in the performance of mean returns and in the volatility of returns.

Insert Tables 4 and 5 here.

#### 2.3 Operating performance

Stock returns are forward-looking and incorporate information quickly and our tests above make use of that feature of capital markets. Accounting numbers are slower at incorporating information, especially as the effects from the pandemic keep unfolding. Here, we conduct a somewhat preliminary look at accounting performance metrics as they respond to the COVID-19 pandemic.

We measure the change in operating performance from the fourth quarter of 2019 to the first quarter of 2020 using three different metrics. *ROA* is the return on assets, calculated as operating income before depreciation divided by book value of assets. *OPM* is the operating profit margin, calculated as operating income before depreciation divided by sales. *AT* is the asset turnover, calculated as sales divided by book value of assets. Following Gompers, Ishii, and Metrick (2003), we estimate median regressions using the least absolute deviation method to reduce the impact of large outliers in the accounting metrics. We include Tobin's q to control for value and growth firms. In an alternative specification, we also include cash and leverage as controls. We include industry fixed effects in all regressions. Standard errors are robust to heteroscedasticity and misspecification.

12 We also analyze whether our results on stock returns and volatility can be explained by a lack of trading for ES stocks. We repeat the difference-in-differences regression specifications of Table 3, but with daily stock trading volume as the dependent variable. The results, reported in the Internet Appendix, show that daily trading volume significantly increases for highly ES-rated firms relative to other firms after the February 24 event date, suggesting that investors stepped in to stop the downward slide in prices. Hence, our stock return and volatility results cannot be explained by thin trading.

Table 6 presents the results. In columns 3 and 4, we find that firms with high ES ratings have higher operating profit margins, consistent with predictions from Albuquerque, Koskinen, and Zhang (2019). One standard deviation increase in ES increases the change in *OPM* by 0.46 (2.181 x 0.212), or 6% of the sample mean change. In columns 5 and 6, we find that asset turnover is lower for firms with high ES ratings relative to other firms during the first quarter. High ES firms appear to increase profit margins even as sales decline. It is possible that these firms either increased prices or maintained their high-profit margins despite the decrease in demand for their products, taking advantage of their customer loyalty consistent with work by Luo and Bhattacharya (2009), Servaes and Tamayo (2013), and Albuquerque, Koskinen, and Zhang (2019). We do not find that ES policies affect the return on assets during the first quarter of 2020. This is not surprising since *ROA* is the product of *OPM* and *AT*.

Insert Table 6 here.

### 3. Two Mechanisms of Resiliency

We study two mechanisms that can potentially explain the resiliency of firms with high ES ratings: customer loyalty and investor segmentation. Both mechanisms predict lower systematic risk associated with high ES stocks. Luo and Bhattacharya (2009) and Albuquerque, Koskinen, and Zhang (2019) propose that customers are more loyal to firms with a strong reputation and that credibly pursue ES policies. In Albuquerque, Koskinen, and Zhang (2019), these firms benefit from lower price elasticity of demand to obtain higher profit margins. These higher profit margins lower operating leverage and reduce firms' systematic risk. Intuitively, customer resiliency delivers stock price resiliency. Albuquerque, Koskinen, and Zhang (2019) present direct evidence of this mechanism by showing that changes in *ROA* are less positively correlated with the business cycle for high ES firms. The evidence in Table 6 that the operating profit margin increases for high ES firms relative to other firms is also consistent with this mechanism. We follow Albuquerque, Koskinen, and Zhang (2019) and others in using advertising expenditures to measure customer loyalty. We expect that the stock return effect we find is more pronounced for firms with high advertising expenditures.

The second mechanism adapts the segmented capital markets model of Heinkel, Kraus, and Zechner (2001), where polluting firms are only held by a subset of investors, since ES investors choose not to hold them. The higher systematic risk of polluting firms is linked to their owners' lack of diversification. Similarly to customer loyalty, investor loyalty can contribute to the resiliency of ES stocks. The literature on Sustainable and Responsible Investments (SRI) shows that investors are more loyal, and less sensitive to SRI funds' performance than to conventional mutual funds' performance (Bollen 2007;

Renneboog, Ter Horst, and Zhang 2011). Our proxy for ES investor preferences is constructed using the idea of revealed preference detailed in Section 2.[13] We expect that stocks with investors with a preference for ES have less systematic risk and total risk.

Table 7 displays the results for stock returns. In our tests, we expand the difference-in-differences regressions in Table 3 to a triple interaction between *Post_COVID*, *ES_treatment*, and a dummy indicating the firms in the top quartile of advertising expenditures (in columns 1 and 2), and to a triple interaction between *Post_COVID*, *ES_treatment*, and a dummy indicating the firms in the top quartile of ES investor preference (in columns 3 and 4). In columns 1 and 2, we find positive estimates of the triple interaction linked to advertising expenditures. Column 2 adds firm and day fixed effects to the regression. In both columns, standard errors are clustered by firm and day. Consistent with the predictions from the first mechanism, there is a significant average abnormal return earned by firms with high ES ratings and high advertising expenditures relative to firms with low ES ratings or low advertising expenditures after February 24. The effect is 0.53% in daily returns, which is 76% larger than the effect for low advertising but high ES firms (0.533/0.302 = 1.76). Columns 3 to 4 show positive estimates on the triple interaction of interest linked to ES investor preference.[14] However, the estimates are not statistically significant. Economically, the point estimate on the ES investor preference triple interaction is half of the effect estimated in the triple interaction with advertising expenditures.

Insert Table 7 here.

Taken together, our return analysis shows strong support for the customer loyalty mechanism for resiliency, which is also consistent with the results regarding operating profit margins. We note that the two mechanisms discussed explain why high ES firms have lower market beta, but they do not fully explain the resiliency of ES firms. The reason is that the dependent variable in the tests above is the CAPM-adjusted stock return, which already accounts for differences in firm beta. Therefore, our results suggest that ES firms appear more resilient during the COVID-19 crisis than what investors expected before the crisis (as reflected by the precrisis firm beta). Still, it is also possible that the better performance of CAPM-adjusted returns is due to a decline in betas during the first quarter for high ES firms. Declining betas of ES stocks may be due to expectations that firm cash flows become less risky than low-ES stocks after the crisis, generating the increased loading on ES as shown in Figure 2.

13 We also use an alternative investor preference measure of ES, which is the institutional ownership of a firm by pension funds and endowments. Starks, Venkat, and Zhu (2018) show the long-term investors prefer high ES stocks. We do not find that this measure has any effects.

14 Cella, Ellul, and Giannetti (2013) show that, during market turmoil, such as the COVID-19 stock market crash, institutional investors with longer trading horizons sell their shares to a lesser extent than investors with short-term trading strategies. To the extent that ES investors have long-term trading horizons, we would expect ES stocks to have smaller price declines.

Table 8 reports the results of tests of the two mechanisms of resiliency for stock return volatility. We repeat the difference-in-differences regressions of Table 5 that uses the daily price range as a proxy for volatility. In columns 1 and 2, we find negative estimates of the triple interaction linked to advertising expenditures, but they are not statistically significant. In columns 3 and 4, we find significantly negative estimates on the triple interaction of interest linked to ES investor preference. Consistent with the predictions from the investor preference mechanism, there is a significant lower range-based volatility by firms with high ES ratings and high ES investor preference relative to other firms after February 24. In fact, the reduction in volatility for high ES firms appears to be concentrated in firms with high ES investor preference.

Overall, our results show a strong effect of customer loyalty on stock returns and of investor ES preference on the volatility of stock returns. These results are consistent with the evidence in Gantchev, Giannetti, and Li (2019), who show that both customers and investors can provide market discipline when firms' ES policies are lacking. This paper shows that influencing the behaviors of both consumers and investors is important for firm resiliency.

Insert Table 8 here.

### 4. Robustness

We investigate two alternative explanations for our findings. One alternative explanation is that the oil price decline in the first quarter of 2020 affected particularly firms in the energy sector, which are known to score low in some dimensions of ES. Energy sector firms would then have significantly lower returns and higher volatility relative to other firms. The Internet Appendix shows that the results are even stronger after excluding firms in the energy sector from our sample.

Another alternative explanation for our results is that some businesses, such as utilities, telecommunication, and financial industries, were considered "essential" and kept on operating in a normal fashion. This may have resulted in some resiliency of cash flows and stock returns for these businesses. To examine this explanation, we investigate the effect of ES ratings on stock returns by industry. We use the Fama-French classification for 12 industries. We repeat the regression specification in Table 3, allowing for triple interactions of *Post_COVID* with the *ES_treatment* and a dummy for each of the industries. Figure 3 shows the results. The figure shows that all but one industry display positive point estimates on the interaction between *Post_COVID* and the *ES_treatment*. Five of those estimates are statistically significant. The one negative point estimates is statistically insignificant.

Overall, the figure suggests that our findings are not associated with any particular industry, but encompass most industries. We go one step further to rule out this alternative explanation. It is possible that the *ES_treatment* is not randomly distributed across industries. We then construct an *ES_treatment* within each industry. This way we are exploiting cross-sectional variation in ES within each industry. The results are very similar to those displayed in Figure 3.

Insert Figure 3 here.

We conduct several additional robustness tests. First, we augment the list of firm-level variables in the cross-sectional regressions of quarterly stock returns and quarterly volatility of stock returns with operating leverage and measures of institutional ownership. Operating leverage, calculated as in Albuquerque, Koskinen, and Zhang (2019) and others, leads to a significant drop in the number of observations. Still, our results hold and are quantitatively similar.

Second, we redo the analysis with MSCI's ESG Research database, previously known as KLD. MSCI rates firms on a variety of strengths and concerns on seven attributes: community, diversity, employee relations, environment, product, human rights, and governance. We exclude corporate governance attributes from our analysis to focus on nongovernance aspects of ESG. We measure ES as the difference between the number of strengths and the number of concerns for each firm in 2016, the last year for which data is available. Given that the number of individual concerns and strengths in each attribute varies over time and across firms, we divide the number of strengths (concerns) for each firm-year across all six ES categories by the maximum possible number of strengths (concerns) in all six categories for each firm. We then subtract the scaled concerns from the scaled strengths to obtain our alternative measure, which is bounded between -1 and 1. We find very similar results with the proxy for ES constructed with MSCI ES data as in Albuquerque, Koskinen, and Zhang (2019). While the MSCI ratings are from 2016 (the latest observation available), firm ES ratings are fairly sticky, which may explain the results. Another possible explanation for the similarity in results despite the lag in the measurement of the ES proxy is that investors care about firm reputation and credibility for ES policies and such reputation depends on a multiyear track record of ES performance. See the Internet Appendix for results with alternative ES ratings.

Third, we change the *Post_COVID* to equal one from January 30 onward. January 30 is the day the World Health Organization declares the outbreak a public health emergency. The results corresponding to Table 3 and Tables 5, 7, and 8 are somewhat weaker because the coefficients of interest are smaller, but retain significance at 10% level or higher.

Finally, we consider the separate roles of E and S in ES. Using Refinitiv's scores, we show that the results in the paper are very similar if we use only the E or the S scores. This is perhaps to be expected because the correlation between the two scores is 0.73, and the correlation between the aggregate score ES and either E or S is over 0.91 (untabulated results). Firms appear to invest in both E and S simulataneously, a reality that limits our ability to evaluate their separate contributions.[15] The last component in ESG, the governance score, has only a correlation of 0.52 with the E score and 0.43 with the S score (untabulated). When we rerun our analysis with the G score, we find that the G score explains the cross-section of stock returns, but only if other firm characteristics are not included in the regression. Thus, the results with the G score serve to reassure that our main results are not picking up a good corporate governance effect.

### 5. Conclusion

The first quarter of 2020 was an extraordinary time for U.S. stock markets: first a calm period before the storm, then the fastest collapse ever, followed with a vigorous rally, all related to the unfolding of an unexpected, exogenous, health pandemic. We use this episode to study how ES firm policies conditioned the stock market response of firms. Specifically, we are interested in testing how customer and investor loyalty based theories of ES account for the stock price properties during the first quarter of 2020. We show that stock prices for firms with high ES scores perform much better than the prices for other firms. The stock market performance is especially strong during the market collapse for high ES stocks with high advertising. Operating profit margin of firms with high ES scores increase in the first quarter of 2020 even as sales decline consistent with a customer loyalty mechanism. In addition, the volatility of stock returns is lower for high ES stocks. Firms held by investors with a preference for ES display larger reductions in the volatility of stock returns. The evidence presented in this paper is consistent with the view that increasing the loyalty of both consumers and investors is an important antecedent for the resiliency of ES firms.

Systematic unobservable differences between high and low ES firms unlikely explain our results, since we control for time-invariant unobservable firm effects in our difference-in-differences regressions. However, ES policies could be possibly correlated with time-varying factors that affect firm value. For example, the COVID-19 pandemic, which threatens firms' survival, could have led to increased investor beliefs that consumer demand for quality products will increase in the long run. As a result, there may be other mechanisms, apart from customer and investor loyalty to ES

[15] We also investigate a potential employee channel within S using Refinitiv's Workplace score. The results are similar to the main results in the paper (except that they are weak in the cross-sectional return regressions), which is perhaps not surprising given the high correlation between Workplace score and ES score of 0.78.

policies, that also render the high-ES firms less susceptible to the COVID-19 shock. We leave the examination of these additional questions for later study.

### Table 1. Summary statistics

This table reports the summary statistics (number of observations, mean, standard deviation [SD], and 25th, 50th [median], and 75th percentiles) for all variables. Table A1 in the appendix defines all variables used in the paper.

| Variable | Obs. | Mean | SD | 25% | Median | 75% |
|---|---|---|---|---|---|---|
| Quarterly abnormal return | 2,171 | -22.971 | 42.482 | -39.841 | -17.397 | 2.803 |
| ES | 2,171 | 0.289 | 0.212 | 0.136 | 0.208 | 0.384 |
| Investor-based ES | 2,123 | 0.544 | 0.064 | 0.514 | 0.555 | 0.587 |
| Tobin’s q | 1,971 | 2.268 | 1.882 | 1.098 | 1.545 | 2.600 |
| Size | 1,973 | 7.138 | 1.919 | 6.062 | 7.180 | 8.329 |
| Cash | 1,972 | 0.156 | 0.209 | 0.023 | 0.067 | 0.191 |
| Leverage | 1,959 | 0.321 | 0.231 | 0.118 | 0.307 | 0.463 |
| ROE | 1,971 | -0.022 | 0.691 | -0.002 | 0.092 | 0.158 |
| Advertising | 2,171 | 0.007 | 0.020 | 0.000 | 0.000 | 0.002 |
| Historical volatility | 2,171 | 2.328 | 1.274 | 1.451 | 1.962 | 2.793 |
| Dividend | 1,973 | 1.735 | 2.365 | 0.000 | 0.905 | 2.628 |
| Volatility | 2,171 | 6.128 | 2.954 | 4.446 | 5.452 | 7.037 |
| Idio. volatility | 2,171 | 4.761 | 3.049 | 2.973 | 4.006 | 5.746 |
| ΔROA_qtr | 1,536 | -0.661 | 2.336 | -1.024 | -0.276 | 0.186 |
| ΔOPM_qtr | 1,515 | -7.989 | 66.460 | -8.022 | -1.632 | 1.269 |
| ΔAT_qtr | 1,755 | -1.236 | 3.255 | -2.007 | -0.258 | 0.091 |
| Daily abnormal return | 134,689 | -0.370 | 5.650 | -1.633 | -0.141 | 1.159 |
| Daily price range | 134,689 | 5.978 | 6.625 | 1.933 | 3.774 | 7.726 |

### Table 2. Cross-sectional regressions for quarterly abnormal returns

This table reports the results of regressions of the first quarter 2020 abnormal returns on firms' ES under several specifications: without firm controls (specification 1), with industry fixed effects (specification 2), and with industry fixed effects and firm controls (specification 3). Control variables are winsorized at the 1% level in each tail. Standard errors are heteroscedasticity robust. The regression constant is not reported for brevity. The numbers in parentheses are *t*-statistics. Table A1 in the appendix defines all variables used in the paper. **p* < .1; ***p* < .05; ****p* < .01.

| Dependent variable | (1)<br>Abnormal return | (2)<br>Abnormal return | (3)<br>Abnormal return |
|---|---|---|---|
| ES | 16.568*** | 19.500*** | 8.542** |
| | (4.30) | (5.56) | (2.05) |
| Tobin's q | | | 3.857*** |
| | | | (8.25) |
| Size | | | 3.179*** |
| | | | (4.85) |
| Cash | | | 27.209*** |
| | | | (4.86) |
| Leverage | | | -29.584*** |
| | | | (-7.05) |
| ROE | | | 0.730 |
| | | | (0.49) |
| Advertising | | | -9.797 |
| | | | (-0.24) |
| Historical volatility | | | -4.427*** |
| | | | (-3.62) |
| Dividend | | | -2.378*** |
| | | | (-4.93) |
| Industry FE | No | Yes | Yes |
| Number of firms | 2,171 | 2,171 | 1,958 |
| Adj. $R^2$ | .006 | .229 | .352 |

### Table 3. Difference-in-differences regressions for daily abnormal returns

This table reports the results of a difference-in-differences estimation of daily abnormal returns during the first quarter of 2020. *ES_treatment* equals one for high ES firms, and zero otherwise. *Post_COVID* equals one from February 24 to March 31, 2020, and zero before this period. *Post_fiscal* equals one from March 18 to March 31, 2020, and zero before this period. Firm and day fixed effects are (not) included in Specification 2 (1). Standard errors are clustered by firm and day. The regression constant is not reported for brevity. The numbers in parentheses are *t*-statistics. Table A1 in the appendix defines all variables used in the paper. *$p < .1$; **$p < .05$; ***$p < .01$.

| Dependent variable | (1)<br>Abnormal return | (2)<br>Abnormal return |
|---|---|---|
| *ES_treatment*Post_COVID* | 0.453*** | 0.453*** |
| | (3.06) | (3.03) |
| *ES_treatment*Post_fiscal* | -0.568 | -0.567 |
| | (-0.94) | (-0.94) |
| *ES_treatment* | -0.000 | |
| | (-0.00) | |
| *Post_COVID* | -1.095*** | |
| | (-3.66) | |
| *Post_fiscal* | 1.280 | |
| | (0.99) | |
| Firm FE | No | Yes |
| Day FE | No | Yes |
| Number of firm-days | 134,689 | 134,689 |
| Adj. $R_2$ | .007 | .082 |

### Table 4. Cross-sectional regressions for volatility

This table reports results for cross-sectional regressions of *Volatility* and *Idio. Volatility* during the first quarter of 2020 on firms' ES under several specifications: without firm controls (specifications 1 and 4), with industry fixed effects (specifications 2 and 5), and with industry fixed effects and firm controls (specifications 3 and 6). Control variables are winsorized at the 1% level in each tail. Standard errors are heteroscedasticity robust. The regression constant is not reported for brevity. The numbers in parentheses are *t*-statistics. Table A1 in the appendix defines all variables used in the paper. *$p < .1$; **$p < .05$; ***$p < .01$.

| Dependent variable | (1)<br>Volatility | (2)<br>Volatility | (3)<br>Volatility | (4)<br>Idio. Volatility | (5)<br>Idio. volatility | (6)<br>Idio. volatility |
|---|---|---|---|---|---|---|
| ES | -2.409*** | -2.315*** | -1.374*** | -2.830*** | -2.740*** | -1.568*** |
| | (-9.54) | (-9.66) | (-5.10) | (-11.06) | (-11.31) | (-5.79) |
| Tobin's q | | | -0.158*** | | | -0.165*** |
| | | | (-6.22) | | | (-6.58) |
| Size | | | -0.105** | | | -0.157*** |
| | | | (-2.14) | | | (-3.15) |
| Cash | | | -0.821** | | | -0.622* |
| | | | (-2.46) | | | (-1.95) |
| Leverage | | | 2.648*** | | | 2.856*** |
| | | | (9.49) | | | (10.08) |
| ROE | | | -0.017 | | | -0.083 |
| | | | (-0.22) | | | (-1.09) |
| Advertising | | | -1.814 | | | 1.434 |
| | | | (-0.94) | | | (0.82) |
| Historical volatility | | | 0.747*** | | | 0.786*** |
| | | | (11.36) | | | (12.24) |
| Dividend | | | 0.058 | | | 0.094** |
| | | | (1.55) | | | (2.39) |
| Industry FE | No | Yes | Yes | No | Yes | Yes |
| Number of firms | 2,171 | 2,171 | 1,958 | 2,171 | 2,171 | 1,958 |
| Adj. $R_2$ | .030 | .140 | .282 | .038 | .143 | .301 |

### Table 5. Difference-in-differences regressions for the daily price range

This table reports the results of difference-in-differences estimation for the daily price range during the first quarter of 2020. *ES_treatment* equals one for high ES firms, and zero otherwise. *Post_COVID* equals one from February 24 to March 31, 2020, and zero before this period. *Post_fiscal* equals one from March 18 to March 31, 2020, and zero before this period. Firm and day fixed effects are (not) included in Specification 2 (1). Standard errors are clustered by firm and day. The regression constant is not reported for brevity. The numbers in parentheses are *t*-statistics. Table A1 in the appendix defines all variables used in the paper. **p* < .1; ***p* < .05; ****p* < .01.

| Dependent variable | (1)<br>Daily price range | (2)<br>Daily price range |
|---|---|---|
| *ES_treatment*Post_COVID* | -0.628*** | -0.630*** |
| | (-3.61) | (-3.45) |
| *ES_treatment*Post_fiscal* | -0.613* | -0.614* |
| | (-1.95) | (-1.88) |
| *ES_treatment* | -0.958*** | |
| | (-11.30) | |
| *Post_COVID* | 5.507*** | |
| | (5.86) | |
| *Post_fiscal* | 4.505*** | |
| | (2.79) | |
| Firm FE | No | Yes |
| Day FE | No | Yes |
| Number of firm-days | 134,689 | 134,689 |
| Adj. $R_2$ | .324 | .622 |

### Table 6. Cross-sectional regressions for operating performance

This table reports the results of regressions of the operating performance's quarterly change (the first quarter of 2020 minus the fourth quarter of 2019) on firms' ES. The dependent variables are the quarterly changes of return on assets (specifications 1 and 2), operating profit margin (specifications 3 and 4), and asset turnover (specifications 5 and 6). All variables are winsorized at the 1% level in each tail. Results in this table are based on LAD (least absolute deviation) regressions. All specifications include industry fixed effects. Standard errors are robust to heteroscedasticity and misspecification. The regression constant is not reported for brevity. The numbers in parentheses are *t*-statistics. Table A1 in the appendix defines all variables used in the paper. **p* < .1; ***p* < .05; ****p* < .01.

| | (1) | (2) | (3) | (4) | (5) | (6) |
|---|---|---|---|---|---|---|
| Dependent variable | *ΔROA_qtr* | *ΔROA_qtr* | *ΔOPM_qtr* | *ΔOPM_qtr* | *ΔAT_qtr* | *ΔAT_qtr* |
| ES | -0.046 | -0.020 | 2.210*** | 2.181*** | -0.297** | -0.298* |
| | (-0.45) | (-0.19) | (3.12) | (2.92) | (-2.01) | (-1.91) |
| Tobin's q | -0.052** | -0.045 | 0.127 | 0.167 | 0.004 | -0.008 |
| | (-2.20) | (-1.52) | (1.08) | (1.14) | (0.14) | (-0.27) |
| Cash | | -0.206 | | -0.565 | | 0.477** |
| | | (-0.73) | | (-0.19) | | (1.97) |
| Leverage | | -0.232* | | 0.936 | | -0.064 |
| | | (-1.66) | | (0.81) | | (-0.45) |
| Industry FE | Yes | Yes | Yes | Yes | Yes | Yes |
| Number of firms | 1,536 | 1,529 | 1,515 | 1,508 | 1,755 | 1,744 |
| $R_2$ | .043 | .045 | .008 | .008 | .068 | .069 |

### Table 7. Triple interactions regressions for daily abnormal returns

This table reports the results of triple interactions estimation for daily abnormal returns during the first quarter of 2020 using difference-in-difference-in-differences regressions. *ES_treatment* equals one for high ES firms, and zero otherwise. *Post_COVID* equals one from February 24 to March 31, 2020, and zero before this period. *Post_fiscal* equals one from March 18 to March 31, 2020, and zero before this period. Specifications 1 and 2 (3 and 4) are triple interaction regressions for high advertising (Investor-based ES) firms. Firm and day fixed effects are (not) included in Specifications 2 and 4 (1 and 3). Standard errors are clustered by firm and day. The regression constant is not reported for brevity. The numbers in parentheses are *t*-statistics. Table A1 in the appendix defines all variables used in the paper. **p* < .1; ***p* < .05; ****p* < .01.

| Dependent variable | (1)<br>Abnormal return | (2)<br>Abnormal return | (3)<br>Abnormal return | (4)<br>Abnormal return |
|---|---|---|---|---|
| *ES_treatment*Post_COVID*Advertising_high* | 0.532** | 0.533** | | |
| | (2.35) | (2.33) | | |
| *ES_treatment*Post_fiscal*Advertising_high* | -1.018** | -1.019** | | |
| | (-2.47) | (-2.45) | | |
| *ES_treatment*Post_COVID*InvestorES_high* | | | 0.272 | 0.271 |
| | | | (1.08) | (1.06) |
| *ES_treatment*Post_fiscal*InvestorES_high* | | | 0.125 | 0.127 |
| | | | (0.28) | (0.28) |
| *ES_treatment*Post_COVID* | 0.302** | 0.302** | 0.283* | 0.284* |
| | (2.07) | (2.05) | (1.77) | (1.74) |
| *ES_treatment*Post_fiscal* | -0.292 | -0.292 | -0.417 | -0.418 |
| | (-0.51) | (-0.51) | (-1.08) | (-1.06) |
| All dummies and other possible interactions included | Yes | Yes | Yes | Yes |
| Firm FE | No | Yes | No | Yes |
| Day FE | No | Yes | No | Yes |
| Number of firm-days | 134,689 | 134,689 | 131,654 | 131,654 |
| Adj. $R_2$ | .007 | .082 | .007 | .084 |

### Table 8. Triple interactions regressions for daily price range

This table reports the results of triple interactions estimation for daily price range during the first quarter of 2020 using difference-in-difference-in-differences regressions. *ES_treatment* equals one for high ES firms, and zero otherwise. *Post_COVID* equals one from February 24 to March 31, 2020, and zero before this period. *Post_fiscal* equals one from March 18 to March 31, 2020, and zero before this period. Specifications 1 and 2 (3 and 4) are triple interaction regressions for high Advertising (Investor-based ES) firms. Firm and day fixed effects are (not) included in Specifications 2 and 4 (1 and 3). Standard errors are clustered by firm and day. The regression constant is not reported for brevity. The numbers in parentheses are *t*-statistics. Table A1 in the appendix defines all variables used in the paper. **p* < .1; ***p* < .05; ****p* < .01.

| Dependent variable | (1) Daily price range | (2) Daily price range | (3) Daily price range | (4) Daily price range |
|---|---|---|---|---|
| *ES_treatment*Post_COVID *Advertising_high* | -0.022 | -0.025 | | |
| | (-0.11) | (-0.11) | | |
| *ES_treatment*Post_fiscal*A dvertising_high* | -0.444* | -0.436 | | |
| | (-1.71) | (-1.34) | | |
| *ES_treatment*Post_COVID *InvestorES_high* | | | -0.879*** | -0.875** |
| | | | (-2.70) | (-2.49) |
| *ES_treatment*Post_fiscal* InvestorES_high* | | | -1.105** | -1.101** |
| | | | (-2.59) | (-2.27) |
| *ES_treatment*Post_COVID* | -0.591*** | -0.593*** | -0.007 | -0.011 |
| | (-3.32) | (-3.11) | (-0.05) | (-0.06) |
| *ES_treatment*Post_fiscal* | -0.458 | -0.462 | -0.242 | -0.244 |
| | (-1.43) | (-1.36) | (-1.01) | (-0.87) |
| All dummies and other possible interactions included | Yes | Yes | Yes | Yes |
| Firm FE | No | Yes | No | Yes |
| Day FE | No | Yes | No | Yes |
| Number of firm-days | 134,689 | 134,689 | 131,654 | 131,654 |
| Adj. $R_2$ | .324 | .622 | .330 | .625 |

**Figure 1. S&P 500 during the first quarter of 2020**

This figure plots the stock market path of S&P 500 during the first quarter of 2020. The red lines represent our two event dates.

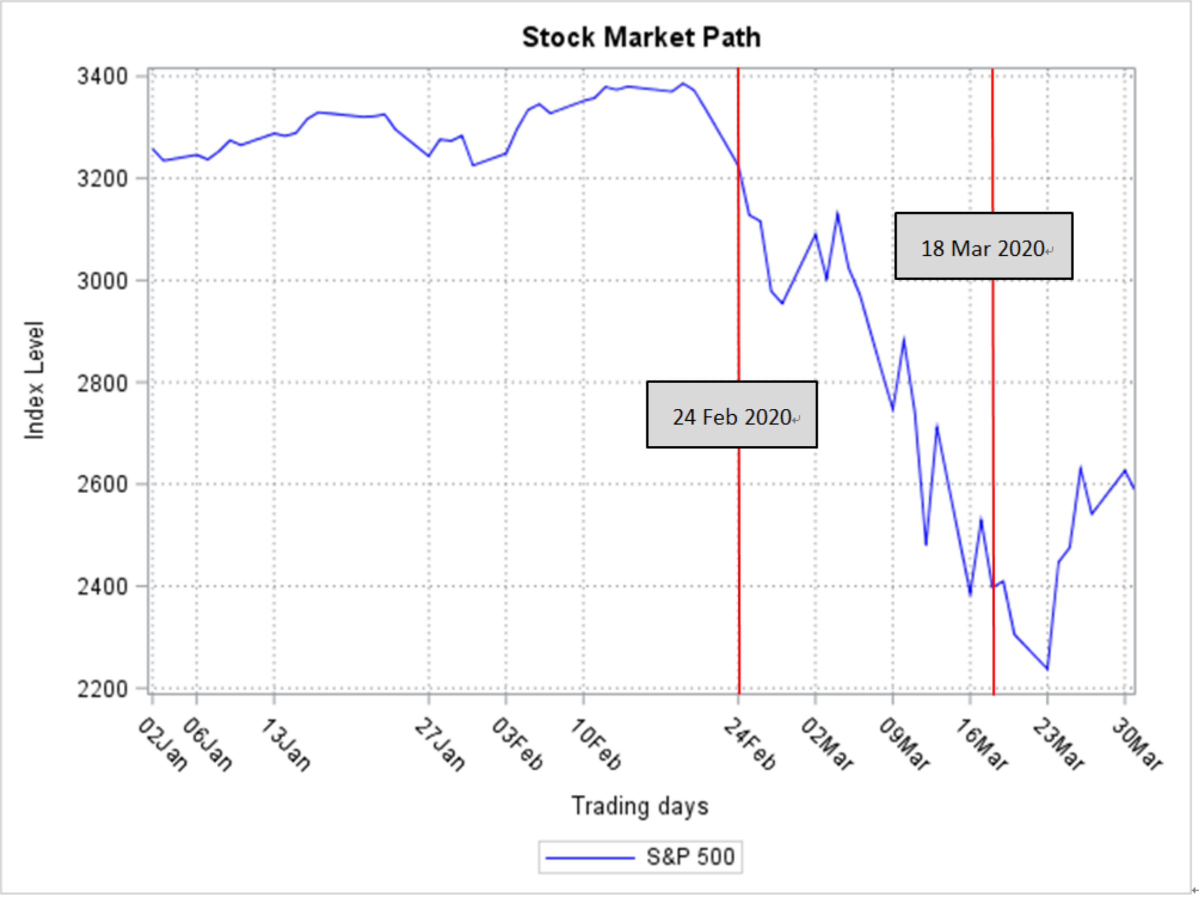

#### Figure 2. Evolution of coefficients from cross-sectional regressions

This figure plots the evolution of coefficients during the first quarter of 2020 from daily cross-sectional regressions of cumulative stock returns (from the start of the quarter to the day) on ES ratings, Tobin's q, firm size, cash to assets, financial leverage, return on equity, advertising expenditures, dividend yield, historical volatility (all lagged 2019 values), and industry fixed effects. It plots the daily loading on ES ratings, cash to assets, and leverage with 90% confidence intervals based on heteroscedasticity-robust standard errors.

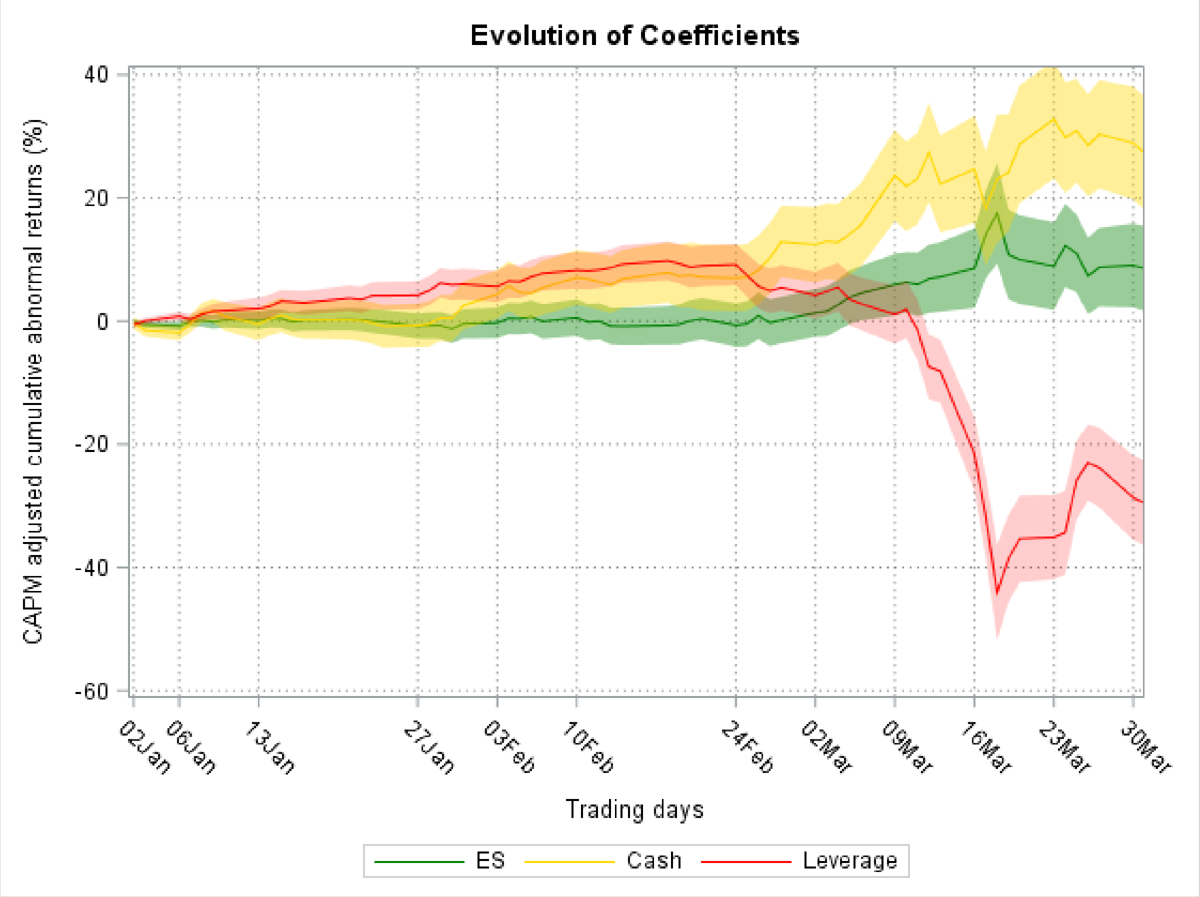

### Figure 3. ES coefficients by industry from triple difference regressions

Regression specification (2) in Table 3 is extended to allow for triple interactions of *Post_COVID* with *ES_treatment* and a dummy for each of the Fama and French 12 industries. The figure plots the point estimates of the triple interaction terms with 90% confidence intervals based on heteroscedasticity-robust standard errors.

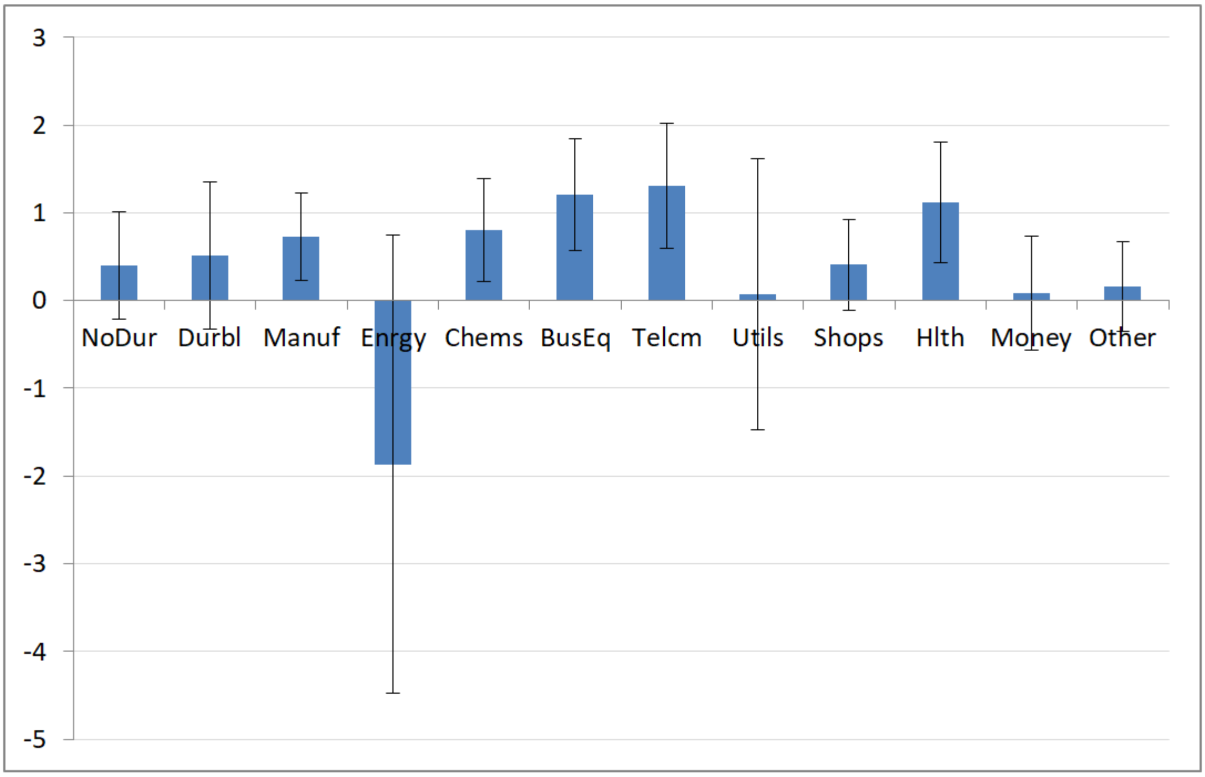

### Appendix
## Table A1. Variables, definitions, and sources

| Variable | Definition | Source |
|---|---|---|
| *ES* | Average between Refinitiv Environment Pillar Score and Social Pillar Score, divided by 100 and measured in 2018. Environment (Social) Pillar Score is the weighted average relative rating of a company based on the reported environmental (social) information and the resultant three (four) environmental (social) category scores. *ES_treatment* is an indicator for firms in the top quartile | Thomson Reuter's Refinitiv ESG |
| *Investor-based ES* | We first measure an investor's revealed ES preference as the value-weighted average *ES* score of its portfolio holdings for each quarter in 2018, and then average across the four quarters. A firm's *Investor-based ES* is the weighted average of its investors' ES based on first quarter 2019 holdings. *InvestorES_high* is an indicator for firms in the top quartile | Our own calculations based on Thomson Reuter's 13F and Refinitiv ESG |
| *Post_COVID* | Dummy variable that equals one from February 24 to March 31, 2020, and zero from January 1 to February 23, 2020 | |
| *Post_fiscal* | Dummy variable that equals one from March 18 to March 31, 2020, and zero from January 1 to 17 to March 17, 2020 | |
| *Tobin's q* | Book value of assets (AT) minus the book value of equity (CEQ) plus the market value of equity (CSHO*PRCC), all divided by book value of assets (AT), measured in $US(2019) | Compustat Annual |
| *Size* | Natural log of firms' sales (SALE) plus one, measured in $US(2019) | Compustat Annual |
| *Cash* | Cash holdings (CHE) over book assets (AT), measured in $US(2019) | Compustat Annual |
| *Leverage* | Book value of debt (DLTT+DLC) over book assets (AT), measured in $US(2019) | Compustat Annual |
| *ROE* | Net income (NI) over book equity (CEQ), measured in $US(2019) | Compustat Annual |
| *Advertising* | Advertising expenditures (XAD) over book assets (AT). Missing values are set to zero, following past literature, measured in $US(2019). *Advertising_high* is an indicator for firms in the top quartile | Compustat Annual |
| *Historical volatility* | Volatility of daily logarithm return (i.e., the logarithm of gross return) of a stock during 2019 | CRSP |
| *Dividend* | Dividend per share (DVPSX) over stock price (PRCC), multiplied by 100, measured in $US(2019) | Compustat Annual |
| *Abnormal return* | The daily *Abnormal return* is the difference between daily logarithm return (i.e., the logarithm of gross return) of a stock and the CAPM beta times the daily logarithm return of the market, expressed as a percentage. The CAPM beta is estimated by using daily returns from 2017 and 2019, where the market index is S&P 500. The quarterly *Abnormal return* is measured over the whole period of the first quarter of 2020, i.e. the difference between the logarithm of the stock's gross quarterly return and the CAPM beta times the logarithm of the market's gross quarterly return | CRSP, Capital IQ North America Daily |
| *Volatility* | Volatility of daily logarithm returns of a stock during the first quarter of 2020 | Capital IQ North America Daily |

| | | |
|---|---|---|
| *Idio. volatility* | Volatility of daily *Abnormal return* of a stock during the first quarter of 2020 | Capital IQ North America Daily |
| *Daily price range* | Daily high-low price range of a stock during the first quarter of 2020, scaled by the midpoint of high and low daily prices. The high price (PRCHD) is the highest trade price for the date. Likewise, the low price (PRCLD) is the lowest trade price for the date | Capital IQ North America Daily |
| *Δ ROA_qtr* | Quarterly change (the first quarter 2020 value minus the fourth quarter 2019 value) in return on assets. Return on assets is operating income before depreciation (OIBDPQ) over book assets (ATQ), multiplied by 100 | Compustat Quarterly |
| *Δ OPM_qtr* | Quarterly change (the first quarter 2020 value minus the fourth quarter 2019 value) in the operating profit margin. Operating profit margin is operating income before depreciation (OIBDPQ) over sales (SALEQ), multiplied by 100 | Compustat Quarterly |
| *Δ AT_qtr* | Quarterly change (the first quarter 2020 value minus the fourth quarter 2019 value) in asset turnover. Asset turnover is sales (SALEQ) over book assets (ATQ), multiplied by 100 | Compustat Quarterly |

## Supplementary Material

cfaa011_Supplementary-data

