# Supplementary material for: Resiliency of Environmental and Social Stocks: An Analysis of the Exogenous COVID-19 Market Crash
Source: Rev Corp Financ Stud. 2020 Jul 7:cfaa011. Online ahead of print. doi: 10.1093/rcfs/cfaa011 (PMC7454887; doi:10.1093/rcfs/cfaa011)
Supplement: cfaa011_Supplementary-data [file cfaa011_supplementary-data.pdf]

For Online Publication:

Internet Appendix of "Resiliency of Environmental and Social Stocks: An Analysis of the Exogenous COVID-19 Market Crash"

Rui Albuquerque
Carroll School of Management, Boston College, ECGI, and CEPR

Yrjo Koskinen
Haskayne School of Business, University of Calgary

Shuai Yang
Haskayne School of Business, University of Calgary

Chendi Zhang
University of Exeter Business School

June 29, 2020

Table A1: Test for the parallel trend assumption

*This table checks the parallel trends assumption by regressing daily abnormal returns from January 1, 2020, to February 23, 2020, on the dummy for high ES firms. ES_treatment equals one for high ES firms, and zero otherwise. Standard errors are clustered by firm and day. The numbers in parentheses are t-statistics. ***, **, and * indicate significance at the 1%, 5%, and 10% levels, respectively. The Appendix contains a detailed description of all the variables.*

	(1)
Dependent variable	Abnormal Return
ES_treatment	-0.000
	(-0.00)
Constant	-0.127*
	(-1.71)
Number of firm-days	76044
Adj. R^2	0.000

Table A2: Difference-in-differences regressions for daily trading volume

*This table reports the results of Difference-in-differences estimation for daily trading volume of stocks during the first quarter of 2020. ES_treatment equals one for high ES firms, and zero otherwise. Post_COVID equals one from 24th February to 31st March 2020, and zero before this period. Post_fiscal equals one from 18th March to 31st March 2020, and zero before this period. Dependent variables are daily trading volume (Capital IQ North America Daily item: CSHTRD) adjusted for stock splits and dividends. CSHTRD is divided by 1 million to reflect daily trading volumes in unit of millions. Firm and day fixed effects are (not) included in Specification 2 (1). Standard errors are clustered by firm and day. The regression constant is not reported for brevity. The numbers in parentheses are t-statistics. ***, **, and * indicate significance at the 1%, 5%, and 10% levels, respectively. The Appendix contains a detailed description of all the variables.*

Dependent variable	(1) Volume	(2) Volume
ES_treatment*Post_COVID	2.026***	2.028***
	(7.19)	(6.71)
ES_treatment*Post_fiscal	0.453*	0.451
	(1.90)	(1.51)
ES_treatment	1.917***	
	(8.17)	
Post_COVID	0.690***	
	(8.34)	
Post_fiscal	0.188	
	(1.59)	
Firm FE	No	Yes
Day FE	No	Yes
Number of firm-days	134688	134688
Adj. R^2	0.077	0.727

Tables A3 – A6: Results with alternative time dummy

Table A3: Difference-in-differences regressions for daily abnormal returns (alternative time dummy)

*This table reports the results of Difference-in-differences estimation of daily abnormal returns during the first quarter of 2020. ES_treatment equals one for high ES firms, and zero otherwise. Post_Jan30 equals one from 30th January to 31st March 2020, and zero before this period. Post_fiscal equals one from 18th March to 31st March 2020, and zero before this period. Firm and day fixed effects are (not) included in Specification 2 (1). Standard errors are clustered by firm and day. The regression constant is not reported for brevity. The numbers in parentheses are t-statistics. ***, **, and * indicate significance at the 1%, 5%, and 10% levels, respectively. The Appendix contains a detailed description of all the variables.*

	(1)	(2)
Dependent variable	Abnormal Return	Abnormal Return
ES_treatment*Post_Jan30	0.227**	0.226**
	(2.20)	(2.17)
ES_treatment*Post_fiscal	-0.346	-0.345
	(-0.58)	(-0.58)
ES_treatment	0.004	
	(0.07)	
Post_Jan30	-0.488**	
	(-2.30)	
Post_fiscal	0.721	
	(0.57)	
Firm FE	No	Yes
Day FE	No	Yes
Number of firm-days	134689	134689
Adj. R^2	0.002	0.082

Table A4: Difference-in-differences regressions for the daily price range (alternative time dummy)

*This table reports the results of Difference-in-differences estimation for the daily price range during the first quarter of 2020. ES_treatment equals one for high ES firms, and zero otherwise. Post_Jan30 equals one from 30th January to 31st March 2020, and zero before this period. Post_fiscal equals one from 18th March to 31st March 2020, and zero before this period. Firm and day fixed effects are (not) included in Specification 2 (1). Standard errors are clustered by firm and day. The regression constant is not reported for brevity. The numbers in parentheses are t-statistics. ***, **, and * indicate significance at the 1%, 5%, and 10% levels, respectively. The Appendix contains a detailed description of all the variables.*

Dependent variable	(1)<br>Daily Price Range	(2)<br>Daily Price Range
ES_treatment*Post_Jan30	-0.263**	-0.264**
	(-2.36)	(-2.22)
ES_treatment*Post_fiscal	-0.939***	-0.942***
	(-3.10)	(-3.00)
ES_treatment	-0.996***	
	(-11.75)	
Post_Jan30	3.032***	
	(4.48)	
Post_fiscal	7.104***	
	(4.80)	
Firm FE	No	Yes
Day FE	No	Yes
Number of firm-days	134689	134689
Adj. R^2	0.242	0.622

Table A5: Triple interactions regressions for daily abnormal returns (alternative time dummy)

*This table reports the results of triple interactions estimation for daily abnormal returns during the first quarter of 2020. ES_treatment equals one for high ES firms, and zero otherwise. Post_Jan30 equals one from 30th January to 31st March 2020, and zero before this period. Post_fiscal equals one from 18th March to 31st March 2020, and zero before this period. Specifications 1 and 2 (3 and 4) are triple interaction regressions for high Advertising (Investor-based ES) firms. Firm and day fixed effects are (not) included in Specifications 2 and 4 (1 and 3). Standard errors are clustered by firm and day. The numbers in parentheses are t-statistics. ***, **, and * indicate significance at the 1%, 5%, and 10% levels, respectively. The Appendix contains a detailed description of all the variables.*

Dependent variable	(1) Abnormal Return	(2) Abnormal Return	(3) Abnormal Return	(4) Abnormal Return
ES_treatment*Post_Jan30*Advertising_High	0.278*	0.278*		
	(1.98)	(1.97)		
ES_treatment*Post_fiscal*Advertising_High	-0.761**	-0.762**		
	(-2.12)	(-2.11)		
ES_treatment*Post_Jan30*InvestorES_High			0.127	0.126
			(0.88)	(0.86)
ES_treatment*Post_fiscal*InvestorES_High			0.262	0.264
			(0.70)	(0.68)
ES_treatment*Post_Jan30	0.157	0.157	0.166	0.166
	(1.54)	(1.52)	(1.59)	(1.57)
ES_treatment*Post_fiscal	-0.146	-0.146	-0.287	-0.287
	(-0.26)	(-0.26)	(-0.82)	(-0.80)
All dummies, constant, and other possible interactions included	Yes	Yes	Yes	Yes
Firm FE	No	Yes	No	Yes
Day FE	No	Yes	No	Yes
Number of firm-days	134689	134689	131654	131654
Adj. R^2	0.002	0.082	0.002	0.083

Table A6: Triple interactions regressions for daily price range (alternative time dummy)

*This table reports the results of triple interactions estimation for daily price range during the first quarter of 2020. ES_treatment equals one for high ES firms, and zero otherwise. Post_Jan30 equals one from 30th January to 31st March 2020, and zero before this period. Post_fiscal equals one from 18th March to 31st March 2020, and zero before this period. Specifications 1 and 2 (3 and 4) are triple interaction regressions for high Advertising (Investor-based ES) firms. Firm and day fixed effects are (not) included in Specifications 2 and 4 (1 and 3). Standard errors are clustered by firm and day. The numbers in parentheses are t-statistics. ***, **, and * indicate significance at the 1%, 5%, and 10% levels, respectively. The Appendix contains a detailed description of all the variables.*

	(1)	(2)	(3)	(4)
Dependent variable	Daily Price Range	Daily Price Range	Daily Price Range	Daily Price Range
ES_treatment*Post_Jan30*Advertising_High	-0.203*	-0.204		
	(-1.85)	(-1.52)		
ES_treatment*Post_fiscal*Advertising_High	-0.383	-0.378		
	(-1.25)	(-1.07)		
ES_treatment*Post_Jan30*InvestorES_High			-0.522***	-0.518**
			(-2.83)	(-2.53)
ES_treatment*Post_fiscal*InvestorES_High			-1.506***	-1.501***
			(-3.36)	(-3.04)
ES_treatment*Post_Jan30	-0.199*	-0.200	0.132	0.130
	(-1.75)	(-1.62)	(1.49)	(1.21)
ES_treatment*Post_fiscal	-0.784**	-0.789**	-0.296	-0.299
	(-2.50)	(-2.39)	(-1.17)	(-1.05)
All dummies, constant, and other possible interactions included	Yes	Yes	Yes	Yes
Firm FE	No	Yes	No	Yes
Day FE	No	Yes	No	Yes
Number of firm-days	134689	134689	131654	131654
Adj. R^2	0.242	0.622	0.248	0.625

Table A7: Industry distribution

This table reports the Fama-French 12 industry distribution of Thomson Reuter's Refinitiv ES sample used in this paper.

Fama-French 12 Industry No.	Fama-French 12 Industry	No. of Firms
1	NoDur	79
2	Durbl	51
3	Manuf	180
4	Enrgy	83
5	Chems	54
6	BusEq	325
7	Telcm	41
8	Utils	54
9	Shops	185
10	Hlth	302
11	Money	556
12	Other	261
Total		2171

Tables A8 – A15: Results excluding energy sector

Table A8: Summary statistics (excluding energy sector)

This table reports the summary statistics (number of observations, mean, standard deviation, 25th, 50th (median) and 75th percentiles) for all variables. The Appendix provides the definition and data sources for all variables.

Variable	Obs.	Mean	Std.Dev.	25%	Median	75%
Quarterly Abnormal Return	2088	-19.768	37.594	-37.181	-15.680	3.433
ES	2088	0.289	0.212	0.137	0.208	0.384
Investor-based ES	2044	0.544	0.063	0.514	0.555	0.587
Tobin's Q	1894	2.312	1.903	1.114	1.581	2.678
Size	1896	7.110	1.925	6.042	7.145	8.311
Cash	1895	0.160	0.211	0.024	0.069	0.196
Leverage	1882	0.320	0.234	0.115	0.306	0.464
ROE	1894	-0.010	0.671	0.006	0.094	0.160
Advertising	2088	0.007	0.020	0.000	0.000	0.002
Historical Volatility	2088	2.286	1.251	1.443	1.923	2.734
Dividend	1896	1.737	2.352	0.000	0.937	2.633
Volatility	2088	5.938	2.485	4.406	5.381	6.873
Idio. Volatility	2088	4.573	2.591	2.929	3.913	5.529
ΔROA_Qtr	1470	-0.570	2.029	-0.960	-0.266	0.184
ΔOPM_Qtr	1449	-6.746	61.277	-7.631	-1.546	1.235
ΔAT_Qtr	1689	-1.248	3.204	-1.967	-0.255	0.079
Daily Abnormal Return	129549	-0.319	5.249	-1.568	-0.122	1.155
Daily Price Range	129549	5.819	6.367	1.898	3.688	7.549

Table A9: Cross-sectional regressions for quarterly abnormal returns (excluding energy sector)

*This table reports the results of regressions of the first quarter 2020 abnormal returns on firms' ES under several specifications: without firm controls (specification 1), with industry fixed effects (specification 2), and with industry fixed effects and firm controls (specification 3). Control variables are winsorized at the 1% level in each tail. Standard errors are heteroskedasticity-robust. The regression constant is not reported for brevity. The numbers in parentheses are t-statistics. ***, **, and * indicate significance at the 1%, 5%, and 10% levels, respectively. The Appendix contains a detailed description of all the variables.*

	(1)	(2)	(3)
Dependent variable	Abnormal Return	Abnormal Return	Abnormal Return
ES	12.856***	16.594***	8.630**
	(3.68)	(4.84)	(2.07)
Tobin's Q			3.837***
			(8.31)
Size			2.653***
			(4.33)
Cash			22.851***
			(4.18)
Leverage			-29.140***
			(-7.08)
ROE			1.407
			(0.93)
Advertising			-13.077
			(-0.32)
Historical Volatility			-3.761***
			(-3.12)
Dividend			-2.879***
			(-5.99)
Industry FE	No	Yes	Yes
Number of firms	2088	2088	1881
Adj. R^2	0.005	0.111	0.264

Table A10: Difference-in-differences regressions for daily abnormal returns (excluding energy sector)

*This table reports the results of Difference-in-differences estimation of daily abnormal returns during the first quarter of 2020. ES_treatment equals one for high ES firms, and zero otherwise. Post_COVID equals one from 24th February to 31st March 2020, and zero before this period. Post_fiscal equals one from 18th March to 31st March 2020, and zero before this period. Firm and day fixed effects are (not) included in Specification 2 (1). Standard errors are clustered by firm and day. The regression constant is not reported for brevity. The numbers in parentheses are t-statistics. ***, **, and * indicate significance at the 1%, 5%, and 10% levels, respectively. The Appendix contains a detailed description of all the variables.*

Dependent variable	(1)<br>Abnormal Return	(2)<br>Abnormal Return
ES_treatment*Post_COVID	0.494***	0.495***
	(3.10)	(3.08)
ES_treatment*Post_fiscal	-0.659	-0.659
	(-1.09)	(-1.09)
ES_treatment	-0.013	
	(-0.33)	
Post_COVID	-1.041***	
	(-3.56)	
Post_fiscal	1.252	
	(0.98)	
Firm FE	No	Yes
Day FE	No	Yes
Number of firm-days	129549	129549
Adj. R^2	0.007	0.089

Table A11: Cross-sectional regressions for volatility (excluding energy sector)

*This table reports results for cross-sectional regressions of Volatility and Idio. Volatility during the first quarter of 2020 on firms' ES under several specifications: without firm controls (specifications 1 and 4), with industry fixed effects (specifications 2 and 5), and with industry fixed effects and firm controls (specifications 3 and 6). Control variables are winsorized at the 1% level in each tail. Standard errors are heteroskedasticity-robust. The regression constant is not reported for brevity. The numbers in parentheses are t-statistics. ***, **, and * indicate significance at the 1%, 5%, and 10% levels, respectively. The Appendix contains a detailed description of all the variables.*

	(1)	(2)	(3)	(4)	(5)	(6)
Dependent variable	Volatility	Volatility	Volatility	Idio. Volatility	Idio. Volatility	Idio. Volatility
ES	-2.126***	-2.062***	-1.341***	-2.521***	-2.460***	-1.533***
	(-10.00)	(-9.67)	(-5.06)	(-11.61)	(-11.46)	(-5.73)
Tobin's Q			-0.154***			-0.161***
			(-6.27)			(-6.67)
Size			-0.070*			-0.117***
			(-1.81)			(-3.03)
Cash			-0.502*			-0.269
			(-1.66)			(-0.95)
Leverage			2.653***			2.873***
			(10.20)			(10.87)
ROE			-0.061			-0.124*
			(-0.91)			(-1.87)
Advertising			-1.761			1.469
			(-0.92)			(0.84)
Historical Volatility			0.709***			0.745***
			(11.77)			(12.56)
Dividend			0.087**			0.122***
			(2.29)			(3.06)
Industry FE	No	Yes	Yes	No	Yes	Yes
Number of firms	2088	2088	1881	2088	2088	1881
Adj. R^2	0.032	0.047	0.238	0.042	0.058	0.271

Table A12: Difference-in-differences regressions for the daily price range (excluding energy sector)

*This table reports the results of Difference-in-differences estimation for the daily price range during the first quarter of 2020. ES_treatment equals one for high ES firms, and zero otherwise. Post_COVID equals one from 24th February to 31st March 2020, and zero before this period. Post_fiscal equals one from 18th March to 31st March 2020, and zero before this period. Firm and day fixed effects are (not) included in Specification 2 (1). Standard errors are clustered by firm and day. The regression constant is not reported for brevity. The numbers in parentheses are t-statistics. ***, **, and * indicate significance at the 1%, 5%, and 10% levels, respectively. The Appendix contains a detailed description of all the variables.*

Dependent variable	(1)<br>Daily Price Range	(2)<br>Daily Price Range
ES_treatment*Post_COVID	-0.582***	-0.587***
	(-3.57)	(-3.40)
ES_treatment*Post_fiscal	-0.697**	-0.698**
	(-2.25)	(-2.15)
ES_treatment	-0.904***	
	(-10.82)	
Post_COVID	5.289***	
	(5.77)	
Post_fiscal	4.651***	
	(2.89)	
Firm FE	No	Yes
Day FE	No	Yes
Number of firm-days	129549	129549
Adj. R^2	0.339	0.636

Table A13: Cross-sectional regressions for operating performance (excluding energy sector)

*This table reports the results of regressions of the operating performance's quarterly change (the first quarter of 2020 minus the fourth quarter of 2019) on firms' ES. The dependent variables are the quarterly changes of return on assets (specifications 1 and 2), operating profit margin (specifications 3 and 4) and asset turnover (specifications 5 and 6). Dependent variables and control variables are winsorized at the 1% level in each tail. Results in this table are based on LAD (least absolute deviation) regressions. All specifications include industry fixed effects. Standard errors are robust to heteroskedasticity and misspecification. The regression constant is not reported for brevity. The numbers in parentheses are t-statistics. ***, **, and * indicate significance at the 1%, 5%, and 10% levels, respectively. The Appendix contains a detailed description of all the variables.*

	(1)	(2)	(3)	(4)	(5)	(6)
Dependent variable	ΔROA_Qtr	ΔROA_Qtr	ΔOPM_Qtr	ΔOPM_Qtr	ΔAT_Qtr	ΔAT_Qtr
ES	-0.059	-0.040	2.172***	2.110***	-0.282**	-0.276*
	(-0.58)	(-0.38)	(3.11)	(2.98)	(-1.96)	(-1.80)
Tobin's Q	-0.051*	-0.044	0.126	0.170	0.005	-0.008
	(-1.95)	(-1.52)	(1.04)	(1.16)	(0.16)	(-0.26)
Cash		-0.218		-0.949		0.448*
		(-0.72)		(-0.32)		(1.69)
Leverage		-0.229*		0.628		-0.073
		(-1.68)		(0.58)		(-0.51)
Industry FE	Yes	Yes	Yes	Yes	Yes	Yes
Number of firms	1470	1463	1449	1442	1689	1678
R^2	0.018	0.021	0.005	0.006	0.073	0.074

Table A14: Triple interactions regressions for daily abnormal returns (excluding energy sector)

*This table reports the results of triple interactions estimation for daily abnormal returns during the first quarter of 2020. ES_treatment equals one for high ES firms, and zero otherwise. Post_COVID equals one from 24th February to 31st March 2020, and zero before this period. Post_fiscal equals one from 18th March to 31st March 2020, and zero before this period. Specifications 1 and 2 (3 and 4) are triple interaction regressions for high Advertising (Investor-based ES) firms. Firm and day fixed effects are (not) included in Specifications 2 and 4 (1 and 3). Standard errors are clustered by firm and day. The numbers in parentheses are t-statistics. ***, **, and * indicate significance at the 1%, 5%, and 10% levels, respectively. The Appendix contains a detailed description of all the variables.*

Dependent variable	(1) Abnormal Return	(2) Abnormal Return	(3) Abnormal Return	(4) Abnormal Return
ES_treatment*Post_COVID*Advertising_High	0.480**	0.480**		
	(2.20)	(2.18)		
ES_treatment*Post_fiscal*Advertising_High	-0.900**	-0.901**		
	(-2.20)	(-2.18)		
ES_treatment*Post_COVID*InvestorES_High			0.322	0.322
			(1.30)	(1.28)
ES_treatment*Post_fiscal*InvestorES_High			0.146	0.147
			(0.31)	(0.30)
ES_treatment*Post_COVID	0.363**	0.363**	0.288*	0.288*
	(2.32)	(2.30)	(1.79)	(1.77)
ES_treatment*Post_fiscal	-0.416	-0.417	-0.458	-0.459
	(-0.74)	(-0.74)	(-1.26)	(-1.23)
All dummies, constant, and other possible interactions included	Yes	Yes	Yes	Yes
Firm FE	No	Yes	No	Yes
Day FE	No	Yes	No	Yes
Number of firm-days	129549	129549	126762	126762
Adj. R^2	0.007	0.090	0.007	0.091

Table A15: Triple interactions regressions for daily price range (excluding energy sector)

*This table reports the results of triple interactions estimation for daily price range during the first quarter of 2020. ES_treatment equals one for high ES firms, and zero otherwise. Post_COVID equals one from 24th February to 31st March 2020, and zero before this period. Post_fiscal equals one from 18th March to 31st March 2020, and zero before this period. Specifications 1 and 2 (3 and 4) are triple interaction regressions for high Advertising (Investor-based ES) firms. Firm and day fixed effects are (not) included in Specifications 2 and 4 (1 and 3). Standard errors are clustered by firm and day. The numbers in parentheses are t-statistics. ***, **, and * indicate significance at the 1%, 5%, and 10% levels, respectively. The Appendix contains a detailed description of all the variables.*

Dependent variable	(1)<br>Daily Price Range	(2)<br>Daily Price Range	(3)<br>Daily Price Range	(4)<br>Daily Price Range
ES_treatment*Post_COVID*Advertising_High	-0.037	-0.038		
	(-0.20)	(-0.18)		
ES_treatment*Post_fiscal*Advertising_High	-0.400	-0.393		
	(-1.59)	(-1.23)		
ES_treatment*Post_COVID*InvestorES_High			-0.939***	-0.936***
			(-2.91)	(-2.69)
ES_treatment*Post_fiscal*InvestorES_High			-1.198**	-1.195**
			(-2.64)	(-2.34)
ES_treatment*Post_COVID	-0.565***	-0.569***	0.005	0.002
	(-3.51)	(-3.27)	(0.04)	(0.01)
ES_treatment*Post_fiscal	-0.535*	-0.537	-0.254	-0.256
	(-1.71)	(-1.62)	(-1.15)	(-0.97)
All dummies, constant, and other possible interactions included	Yes	Yes	Yes	Yes
Firm FE	No	Yes	No	Yes
Day FE	No	Yes	No	Yes
Number of firm-days	129549	129549	126762	126762
Adj. R^2	0.339	0.636	0.344	0.639

Tables A16 – A20: Results using the MSCI ES sample

Table A16: Summary statistics (MSCI ES sample)

This table reports the summary statistics (number of observations, mean, standard deviation, 25th, 50th (median) and 75th percentiles) for all variables. ES-MSCI: we divide the number of strengths (concerns) for each firm-year across all six ES categories excluding governance by the maximum possible number of strengths (concerns) in all six categories for each firm-year, to ensure comparability over time and across firms. We then subtract the scaled concerns from the scaled strengths to obtain a net measure. It is measured in 2016. ES-MSCI_treatment is an indicator for firms in the top quartile. Source: MSCI's ESG Research. The Appendix provides the definition and data sources for all other variables.

Variable	Obs.	Mean	Std.Dev.	25%	Median	75%
Quarterly Abnormal Return	1848	-22.175	40.743	-37.648	-15.824	2.260
ES-MSCI	1848	0.027	0.054	0.000	0.026	0.051
Tobin's Q	1686	2.148	1.677	1.101	1.525	2.428
Size	1688	7.517	1.668	6.489	7.424	8.585
Cash	1687	0.136	0.178	0.022	0.063	0.166
Leverage	1681	0.327	0.229	0.132	0.318	0.461
ROE	1686	0.012	0.646	0.022	0.100	0.167
Advertising	1848	0.007	0.020	0.000	0.000	0.002
Historical Volatility	1848	2.197	1.201	1.401	1.847	2.589
Dividend	1688	1.843	2.335	0.000	1.214	2.743
Volatility	1848	5.973	2.860	4.341	5.250	6.836
Idio. Volatility	1848	4.596	2.953	2.846	3.784	5.423
Daily Abnormal Return	114548	-0.356	5.455	-1.568	-0.134	1.103
Daily Price Range	114548	5.781	6.601	1.840	3.567	7.430

Table A17: Cross-sectional regressions for quarterly abnormal returns (MSCI ES sample)

*This table reports the results of regressions of the first quarter 2020 abnormal returns on firms' ES under several specifications: without firm controls (specification 1), with industry fixed effects (specification 2), and with industry fixed effects and firm controls (specification 3). Control variables are winsorized at the 1% level in each tail. Standard errors are heteroskedasticity-robust. The regression constant is not reported for brevity. The numbers in parentheses are t-statistics. ***, **, and * indicate significance at the 1%, 5%, and 10% levels, respectively. The Appendix contains a detailed description of all the variables.*

	(1)	(2)	(3)
Dependent variable	Abnormal Return	Abnormal Return	Abnormal Return
ES-MSCI	99.499***	75.564***	28.596*
	(6.48)	(5.22)	(1.77)
Tobin's Q			3.854***
			(7.20)
Size			2.077***
			(3.06)
Cash			25.406***
			(4.53)
Leverage			-31.491***
			(-7.32)
ROE			0.401
			(0.25)
Advertising			20.982
			(0.51)
Historical Volatility			-5.961***
			(-4.70)
Dividend			-2.546***
			(-4.86)
Industry FE	No	Yes	Yes
Number of firms	1848	1848	1680
Adj. R^2	0.017	0.194	0.349

Table A18: Difference-in-differences regressions for daily abnormal returns (MSCI ES sample)

*This table reports the results of Difference-in-differences estimation of daily abnormal returns during the first quarter of 2020. ES-MSCI_treatment equals one for high ES firms, and zero otherwise. Post_COVID equals one from 24th February to 31st March 2020, and zero before this period. Post_fiscal equals one from 18th March to 31st March 2020, and zero before this period. Firm and day fixed effects are (not) included in Specification 2 (1). Standard errors are clustered by firm and day. The regression constant is not reported for brevity. The numbers in parentheses are t-statistics. ***, **, and * indicate significance at the 1%, 5%, and 10% levels, respectively. The Appendix contains a detailed description of all the variables.*

Dependent variable	(1)<br>Abnormal Return	(2)<br>Abnormal Return
ES-MSCI_treatment*Post_COVID	0.533***	0.532***
	(3.08)	(3.05)
ES-MSCI_treatment*Post_fiscal	-0.746	-0.744
	(-1.24)	(-1.23)
ES-MSCI_treatment	0.053*	
	(1.72)	
Post_COVID	-1.029***	
	(-3.59)	
Post_fiscal	1.192	
	(0.97)	
Firm FE	No	Yes
Day FE	No	Yes
Number of firm-days	114548	114548
Adj. R^2	0.006	0.074

Table A19: Cross-sectional regressions for volatility (MSCI ES sample)

*This table reports results for cross-sectional regressions of Volatility and Idio. Volatility during the first quarter of 2020 on firms' ES under several specifications: without firm controls (specifications 1 and 4), with industry fixed effects (specifications 2 and 5), and with industry fixed effects and firm controls (specifications 3 and 6). Control variables are winsorized at the 1% level in each tail. Standard errors are heteroskedasticity-robust. The regression constant is not reported for brevity. The numbers in parentheses are t-statistics. ***, **, and * indicate significance at the 1%, 5%, and 10% levels, respectively. The Appendix contains a detailed description of all the variables.*

	(1)	(2)	(3)	(4)	(5)	(6)
Dependent variable	Volatility	Volatility	Volatility	Idio. Volatility	Idio. Volatility	Idio. Volatility
ES-MSCI	-8.529***	-7.228***	-1.900	-9.388***	-8.313***	-2.028*
	(-7.31)	(-6.51)	(-1.56)	(-7.86)	(-7.45)	(-1.67)
Tobin's Q			-0.183***			-0.200***
			(-5.89)			(-6.58)
Size			-0.154***			-0.217***
			(-2.98)			(-4.14)
Cash			-0.932**			-0.661*
			(-2.43)			(-1.79)
Leverage			2.637***			2.853***
			(9.22)			(9.82)
ROE			-0.106			-0.170*
			(-1.26)			(-1.78)
Advertising			-1.648			3.408
			(-0.69)			(1.55)
Historical Volatility			0.839***			0.893***
			(10.55)			(11.48)
Dividend			0.050			0.082*
			(1.24)			(1.95)
Industry FE	No	Yes	Yes	No	Yes	Yes
Number of firms	1848	1848	1680	1848	1848	1680
Adj. R^2	0.026	0.106	0.297	0.029	0.101	0.319

Table A20: Difference-in-differences regressions for the daily price range (MSCI ES sample)

*This table reports the results of Difference-in-differences estimation for the daily price range during the first quarter of 2020. ES-MSCI_treatment equals one for high ES firms, and zero otherwise. Post_COVID equals one from 24th February to 31st March 2020, and zero before this period. Post_fiscal equals one from 18th March to 31st March 2020, and zero before this period. Firm and day fixed effects are (not) included in Specification 2 (1). Standard errors are clustered by firm and day. The regression constant is not reported for brevity. The numbers in parentheses are t-statistics. ***, **, and * indicate significance at the 1%, 5%, and 10% levels, respectively. The Appendix contains a detailed description of all the variables.*

Dependent variable	(1)<br>Daily Price Range	(2)<br>Daily Price Range
ES-MSCI_treatment*Post_COVID	-0.611***	-0.610***
	(-3.58)	(-3.38)
ES-MSCI_treatment*Post_fiscal	-0.706**	-0.705**
	(-2.24)	(-2.14)
ES-MSCI_treatment	-0.766***	
	(-8.14)	
Post_COVID	5.429***	
	(5.86)	
Post_fiscal	4.591***	
	(2.88)	
Firm FE	No	Yes
Day FE	No	Yes
Number of firm-days	114548	114548
Adj. R^2	0.320	0.629
